# Design, Synthesis, Analysis, and Cytotoxicity of Novel Heteroaryl Derivatives of Dipyridothiazines

**DOI:** 10.3390/cimb48020128

**Published:** 2026-01-23

**Authors:** Emilia Martula, Paulina Strzyga-Łach, Marta Struga, Katarzyna Żurawska, Weronika Bagrowska, Anna Kasprzycka, Małgorzata Jeleń, Beata Morak-Młodawska

**Affiliations:** 1 Doctoral School, The Medical University of Silesia, 40-055 Katowice, Poland; emilia.martula2903@gmail.com; 2 Department of Organic Chemistry, Faculty of Pharmaceutical Sciences, The Medical University of Silesia, Jagiellońska 4, 41-200 Sosnowiec, Poland; manowak@sum.edu.pl; 3 Department of Biochemistry, Medical University of Warsaw, 02-097 Warsaw, Poland; paulina.strzyga-lach@wum.edu.pl (P.S.-Ł.); marta.struga@wum.edu.pl (M.S.); 4 Biotechnology Centre, The Silesian University of Technology, Krzywoustego Street 8, 44-100 Gliwice, Poland; katarzyna.hopko@polsl.pl (K.Ż.); anna.kasprzycka@polsl.pl (A.K.); 5 Tunneling Group, Biotechnology Centre, The Silesian University of Technology, Krzywoustego 8, 44-100 Gliwice, Poland; weronika.bagrowska@polsl.pl; 6 Department of Organic Chemistry, Bioorganic Chemistry and Biotechnology, Faculty of Chemistry, The Silesian University of Technology, Krzywoustego Street 4, 44-100 Gliwice, Poland

**Keywords:** heterocycles, diazaphenothiazines, cytotoxicity, anticancer activity, molecular docking, histone deacetylase 6 (HDAC6)

## Abstract

Heterocyclic compounds have enormous pharmacological potential and therefore play a key role in the design of new drugs. Dipyridothiazines, both heterocyclic compounds and phenothiazine derivatives, exhibit promising anticancer, immunostimulatory, and antioxidant activities. The aim of this study was to design, synthesize, and evaluate the cytotoxicity of new 10-heteroaryl dipyridothiazines based on 2,7- and 3,6-diazaphenothiazine cores. The structural characterization of the new compounds was confirmed by spectroscopic methods. Cytotoxicity analysis was performed using the MTT assay against human keratinocytes (HaCaT) and two types of cancer cell lines: breast cancer (MDA-MB-231), lung carcer (A-549). The reference drugs used in the study were doxorubicin and cisplatin. The group of derivatives studied included active compounds as well as inactive derivatives. In order to explain differences in an activity level, molecular modelling supported by molecular dynamics was performed on histone deacetylase 6 (HDAC6), a known therapeutic target associated with oncogenic transformation and cancer metastasis. Molecular docking indicated that the derivative formed on the 2,7-diazaphenothiazine core is a more potent HDAC6 inhibitor, characterized by more stable binding and more favourable complex energy, despite minimal structural differences compared to the compound formed on the 3,6-diazaphenothiazine core. A preliminary SAR analysis was performed.

## 1. Introduction

Heterocyclic compounds are cyclic organic structures in which one or more carbon atoms are replaced by heteroatoms such as nitrogen, sulfur, or oxygen. Due to their remarkable structural diversity and varied physicochemical properties, heterocycles have been extensively studied and are widely described in the scientific literature [1,2,3,4,5,6]. These characteristics make them valuable frameworks for the rational design of novel biologically active molecules.

Heterocyclic systems can be found in many existing drugs as well as newly obtained compounds whose pharmacological properties are being studied [7,8,9,10]. Sulfur-containing heterocycles constitute one of the most important classes of heterocyclic compounds that are routinely used in chemistry. Such derivatives occur in both natural compounds and pharmaceuticals [9]. The most common sulfur-containing heterocycles used in drug design have five- and six-membered rings and include thiazole, isothiazole, thiophene, thiopyran, thiazolidine, and thiazepine [3]. These heterocycles have documented anticancer, antiviral, anti-inflammatory, antimicrobial, and antituberculosis activity. More importantly, several FDA-approved drugs belonging to the sulfur heterocycle group are available on the market. These include raloxifene [11], a therapeutic agent used in the management of breast cancer; ritonavir [12], a potent antiviral drug; rosiglitazone [13], a compound clinically used for diabetes therapy; and thiabendazole [14], an antifungal drug. The second major group of heterocycles are compounds containing a nitrogen atom in their structure [5]. The most well-known heterocycles of this type include pyridine, quinoline, pyrrole, benzimidazole, benzothiazoles, and benzoxazoles. These compounds occur both naturally and synthetically. Nitrogen heterocyclic compounds are associated with a wide range of pharmacological activities, encompassing antitubercular, anticancer, antiviral, antimalarial, antileishmanial, anti-inflammatory, and anti-Alzheimer’s disease activities [15,16,17].

Phenothiazines constitute a distinct class of sulfur–nitrogen heterocycles that are entirely synthetic, with no known natural analogues or biosynthetic precursors [18,19]. Their unique tricyclic framework has contributed to broad pharmacological relevance, making phenothiazines an important scaffold in medicinal chemistry. Chemically, they are tricyclic systems composed of two benzene rings connected by nitrogen and sulfur atoms at the 1,4-position. These compounds were introduced into psychiatry as neuroleptic drugs in the 1950s, demonstrating strong affinity for dopaminergic receptors. Furthermore, drugs from this group exhibited valuable sedative, anxiolytic, and antiemetic activities. Moreover, the structure of phenothiazines has undergone numerous modifications aimed at obtaining new derivatives with pharmacological properties targeted at contemporary lifestyle diseases [19].

**Figure 1:**
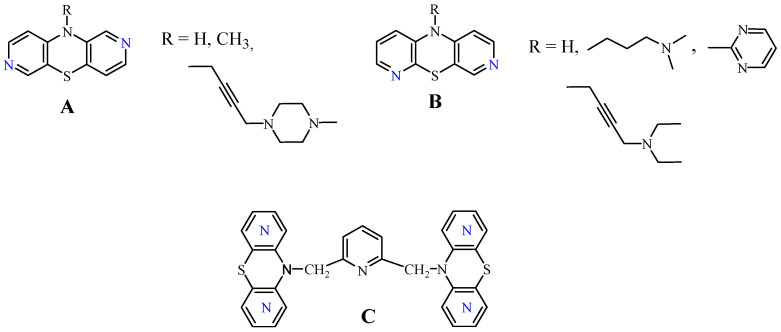
Selected dipyridothiazine derivatives with anticancer activity [20,24,25,28]. Structures were drawn with ChemDraw (Version 23.1.1.3). RevvitySignalsSoftware (2024), Waltham, MA, USA.

Modified phenothiazine systems include dipyridothiazines, whose chemical structure contains pyridine rings instead of two benzene rings. Several compounds from this group have demonstrated valuable anticancer, antioxidant, and immunomodulatory properties in a variety of in vitro studies [20]. Pharmacokinetic analyses have shown that these compounds exhibit moderate lipophilicity and meet Lipinski’s Rule of Five, confirming their bioavailability [21,22,23,24]. Selected derivatives from this class, encompassing both 2,7- and 3,6-diazaphenothiazine frameworks, have previously exhibited pronounced anticancer activity against glioma, melanoma, breast cancer, ovarian cancer, lung cancer and skin cancer. Moreover, these compounds demonstrated immunosuppressive effects in both in vitro and in vivo models, as well as antioxidant properties [20,25,26,27,28,29]. Representative anticancer-active derivatives from this group (**A**, **B**, and **C**) are presented in Figure 1. The observed biological activity was strongly dependent on the nature of the substituent at the thiazine nitrogen atom—including alkyl and heteroaryl groups (e.g., 2-pyrimidyl), alkylaminoalkyl or alkylaminobutynyl moieties, or dimeric systems linking thiazine units—as well as on the positional arrangement of nitrogen atoms within the dipyridothiazine core. Furthermore, these compounds exhibited low cytotoxicity toward normal cells within the tested concentration range, indicating a favourable selectivity profile [20,25,26]. The observed anticancer effects were mechanistically linked to the activation of the mitochondrial apoptotic pathway, accompanied by a disruption of the balance between the antiapoptotic BCL-2 and proapoptotic BAX proteins. In addition, the cytotoxic activity of these derivatives was associated with partial DNA intercalation, which may contribute to their ability to impair cancer cell proliferation [25,26].

Among the evaluated compounds, the 3,6-diazaphenothiazine derivative bearing a 2-pyrimidyl moiety (**B**) emerged as particularly effective. It exhibited marked selectivity against MCF-7 breast cancer cells, with an IC_50_ of 0.73 μg/mL. qPCR-based mechanistic studies indicated that its antiproliferative activity involves induction of the mitochondrial apoptotic pathway [25]. In later work, we demonstrated that structurally related dipyridothiazine dimers (**C**) also display anticancer properties, which may be linked to inhibition of histone deacetylase activity [28,29]. Moreover, studies reported by Vögerl et al. demonstrated that phenothiazine derivatives may act as selective inhibitors of histone deacetylase 6, thereby exerting the desired cytotoxic effects [30]. It is worth noting here that among all histone deacetylases, histone deacetylase 6 (HDAC6) is a key target for drug design due to its role in oncogenic transformation and metastasis [31,32]. Histone deacetylase 6 (HDAC6) plays a significant role in the pathogenesis of many cancers, primarily by regulating processes not directly related to chromatin. It regulates cancer cell proliferation, viability, migration, and invasion. Due to its specific localization and functions, HDAC6 is considered an attractive cancer therapeutic target [33,34].

Based on the documented anticancer potential of dipyridothiazines, including derivatives bearing a 2-pyrimidyl substituent, and the well-established pharmacological relevance of heterocyclic scaffolds, we designed and synthesized a new series of heteroaryl dipyridothiazines incorporating chlorinated azine rings (pyrazine and pyrimidine). This study aimed to assess the impact of a strongly electronegative substituent within the heteroaryl moiety, as well as the effect of nitrogen atom positioning, on the resulting biological activity. The challenge of our research was to develop an effective synthesis of new compounds, document their structures using spectroscopic methods, and determine their cytotoxicity against selected cells: a breast cancer cell line (MDA-MB-231), a lung cancer cell line (A-549), and normal human keratinocytes (HaCaT). To analyze and explain the differences in anticancer activity in silico, preliminary molecular docking studies targeting histone deacetylase 6 were performed.

## 2. Materials and Methods

### 2.1. Chemical Part

Melting points were measured in open capillaries on a Boetius apparatus (Stuart Equipment, Stone, UK) according to the literature recommendations described in reference [35]. ^1^H NMR spectroscopic analyses were obtained using a Bruker AscendTM 600 spectrometer operating at 600 MHz (Bruker, Rheinstetten, Germany). Samples were measured in a deuterated DMSO solution. ^13^C NMR spectra were recorded at 150 MHz on a Bruker AscendTM 600 spectrometer [36]. Mass spectrometry spectra were obtained using the Electro Spray Ionization (ESI) technique as high-resolution spectra (HR MS) on a Brucker Impact II instrument (Bruker, Billerica, MA, USA). The commercial reagents and solvents used in the syntheses include 2,3-dichloropyrazine (**a**), 2,5-dichloropyrimidine (**b**), 2,6-dichloropyrazine (**c**) (Sigma-Aldrich, Burlington, MA, USA), sodium hydroxide (Sigma-Aldrich), DMF, ethanol, and chloroform (POCh, Gliwice, Poland).

The parent dipyridothiazines (compounds **1** and **2**), which served as building blocks, were obtained according to the literature [20,25].

### 2.2. General Procedure for Synthesis of 10 Substituted Diazaphenothiazine Derivatives (***3***–***8***)

A mixture of 10H-dipyridothiazine (1 or 2; 0.5 mmol, 0.100 g) and NaH (0.030 g, 60% dispersion in mineral oil) in anhydrous DMF (10 mL) was stirred at room temperature for 30 min. The appropriate dichlorodiazine derivative (**a**–**c**; 0.5 mmol, 0.0745 g) was then added, and the reaction mixture was stirred for 24 h. The mixture was poured into water (30 mL), and the precipitate was filtered off. The filtrate was extracted with CHCl_3_ (three times using 30 mL of solvent each time), and the combined organic layers were dried over Na_2_SO_4_ and concentrated under reduced pressure. The crude products were purified by column chromatography on Al_2_O_3_ using CHCl_3_ as an eluent to afford the corresponding derivatives:

- 10-(3-chloropyrazin-2-yl)-10H-dipyrido[3,4-b:3′,4′-e][1,4]thiazine (**3**) (131.8 mg; 84%), m.p. 132–134 °C
  ^1^H NMR (DMSO_d6_) δ (ppm): 6.51 (d, *J* = 5.4 Hz, 1H), 6.94 (d, *J* = 5.1 Hz, 1H), 7.76 (s, 1H), 7.84–7.87 (m, 2H), 7.97 (d, *J* = 5.1 Hz, 1H), 8.55 (s, 2H_Pyrazin_).
  ^13^C NMR (DMSO_d6_) δ (ppm): 109.6, 111.9, 121.1, 127.3, 135.7, 136.2, 143.5, 143.5, 144.2, 146.2, 146.9, 147.8, 149.8.
  HRMS (EI) *m*/*z* for [C_14_H_8_ClN_5_S + H] calc. 314.0262 Found: 314.0263 (100%), 316.0235 (35%).
- 10-(5-chloropyrimidin-2-yl)-10H-dipyrido[3,4-b:3′,4′-e][1,4]thiazine (**4**) (139.6 mg; 89%), m.p. 172–275 °C
  ^1^H NMR (DMSO_d6_) δ (ppm): 6.52 (d, *J* = 5.1 Hz, 1H), 6.96 (d, *J* = 5.1 Hz, 1H), 7.78 (s, 1H), 7.86–7.88 (m, 1H), 7.98 (d, *J* = 5.1 Hz, 2H), 8.94 (s, 2H_Pyrazin_).
  ^13^C NMR (DMSO_d6_) δ (ppm): 109.7, 112.0, 121.5, 127.3, 130.7, 135.7, 136.3, 144.3, 146.3, 147.8, 149.8, 158.4, 159.3.
  HRMS (EI) *m*/*z* for [C_14_H_8_ClN_5_S + H] calc. 314.0262 Found: 314.0259 (100%), 316.0228 (41%).
- 10-(6-chloropyrazin-2-yl)-10H-dipyrido[3,4-b:3′,4′-e][1,4]thiazine (**5**) (125.5 mg; 80%), m.p. 117–119 °C
  ^1^H NMR (DMSO_d6_) δ (ppm): 6.52 (d, *J* = 5.1 Hz, 1H), 6.95 (d, *J* = 5.1 Hz, 1H), 7.72 (s, 1H), 7.86–7.88 (m, 2H), 7.98 (d, *J* = 5.1 Hz, 1H), 8.85 (s, 2H_Pyrazin_).
  ^13^C NMR (DMSO_d6_) δ (ppm): 109.7, 111.9, 121.5, 127.3, 135.7, 136.3, 143.6, 144.3, 146.30, 147.0, 147.8, 149.8.
  HRMS (EI) *m*/*z* for [C_14_H_8_ClN_5_S + H] calc. 314.0262 Found: 314.0271 (100%), 316.0232 (57%).
- 10-(3-chloropyrazin-2-yl)-10H-dipyrido[2,3-b:4′,3′-e][1,4]thiazine (**6**) (133.3 mg; 85%), m.p. 98–100 °C
  ^1^H NMR (DMSO_d6_) δ (ppm): 6.48 (m, 1H), 6.85 (m, 1H), 6.96 (s, 1H), 7.80–7.85 (m, 1H), 7.86 (s, 1H), 7.96 (s, 1H), 8.56 (s, 1H_Pyrazin_), 9.14 (s, 1H_Pyrazin_).
  ^13^C NMR (DMSO_d6_) δ (ppm): 109.2, 114.0, 121.1, 123.3, 136.3, 140.5, 143.3, 143.6, 146.3, 146.5, 146.9, 149.5.
  HRMS (EI) *m*/*z* for [C_14_H_8_ClN_5_S + H] calc. 314.0262 Found: 314.0270 (100%), 316.0231 (55%).
- 10-(5-chloropyrimidin-2-yl)-10H-dipyrido[2,3-b:4′,3′-e][1,4]thiazine (**7**) (141.2 mg; 91%), m.p. 286–288 °C
  ^1^H NMR (DMSO_d6_) δ: 6.48 (d, *J* = 5.4 Hz, 1H), 6.85 (d, *J* = 7.8 Hz, 1H), 6.95–6.97 (m, 1H), 7.80 (d, *J* = 4.2 Hz, 1H), 7.86 (s, 1H), 7.96 (d, *J* = 5.4 Hz, 1H), 8.95 (s, 1H_Pyrazin_), 9.14 (s, 1H_Pyrazin_).
  ^13^C NMR (DMSO_d6_) δ (ppm): 109.2, 114.0, 121.1, 123.3, 130.7, 136.3, 140.5, 143.3, 146.2, 146.5, 149.5, 158.4, 159.3.
  HRMS (EI) *m*/*z* for [C_14_H_8_ClN_5_S + H] calc. 314.0262 Found: 314.0270, (100%), 316.0231 (58.0%).
- 10-(6-chloropyrazin-2-yl)-10H-dipyrido[2,3-b:4′,3′-e][1,4]thiazine (**8**) (128.6 mg; 82%), m.p. 234–236 °C
  ^1^H NMR (DMSO_d6_) δ (ppm): 6.49 (d, *J* = 5.4 Hz, 1H), 6.85 (d, *J* = 7.8 Hz, 1H), 6.95–6.97 (m, 1H), 7.80 (d, *J* = 4.2 Hz, 1H), 7.86 (s, 1H), 7.96 (d, *J* = 4.8 Hz, 1H), 8.56 (s, 1H_Pyrazin_), 9.14 (s, 1H_Pyrazin_).
  ^13^C NMR (DMSO_d6_) δ (ppm): 109.2, 114.1, 121.0, 123.3, 136.2, 140.5, 143.3, 143.6, 146.2, 146.5, 147.0, 149.5.
  HRMS (EI) *m*/*z* for [C_14_H_8_ClN_5_S + H] calc. 314.0262 Found: 314.0271 (100%), 316.0232 (58.2%).

### 2.3. Biological Evaluation

#### 2.3.1. Cell Line and Culture

The cell lines used in this study—human breast adenocarcinoma (MDA-MB-231), human lung cancer (A-549), and immortal HaCaT keratinocytes—were obtained from the American Type Culture Collection (ATCC, Rockville, MD, USA), ensuring their verified origin and adherence to reference characteristics. Each line was maintained according to ATCC-recommended specifications for its cell type. All cultures were grown in standard Dulbecco’s Modified Eagle Medium (DMEM), a commonly used medium for culturing adherent cells. The medium was supplemented with 10% fetal bovine serum (FBS), serving as a source of growth factors and nutrients, as well as the antibiotics penicillin (100 U/mL) and streptomycin (100 μg/mL), which reduced the risk of accidental bacterial infections. Cells were maintained under conditions typical of mammalian cell lines—a humidified atmosphere, 5% CO_2_, and a physiological temperature of 37 °C. Stable incubation conditions ensured optimal metabolic processes and proper cell proliferation. Cells were transferred to new vessels when the monolayer reached approximately 80–90% surface coverage. A 0.25% trypsin solution from Gibco Life Technologies (Grand Island, NY, USA) was used to detach the cells from the substrate, allowing for the safe loosening of cell connections and preparation of a suspension for further culture.

#### 2.3.2. MTT Cell Viability Assay

Cell viability was assessed using the MTT assay, in which 3-(4,5-dimethylthiazol-2-yl)-2,5-diphenyltetrazolium bromide (MTT) is reduced by mitochondrial dehydrogenases present only in living cells, leading to the formation of insoluble formazan [37]. Cells were distributed in microplates of appropriate density (96-well plates at a density of 1 × 10^4^ cell), allowing for stabilization before exposure to varying concentrations of the test compounds. Control samples included untreated cells. After incubation (24 h at 37 °C in a CO_2_ environment), the MTT reagent was introduced, allowing for the formation of a coloured reaction product; then the formazan was dissolved (isopropanol–DMSO mixture (1:1)) for spectrophotometric measurement at the appropriate wavelength (570 nm using a UVM 340 reader (ASYS Hitech GmbH, Seekirchen am Wallersee, Austria). The obtained absorbance values formed the basis for determining cytotoxicity parameters, including the determination of IC_50_ values using the GraphPad Prism 8 software (GraphPad Software).

### 2.4. The Statistical Analysis

The GraphPad Prism 9 software [38] (GraphPad Software, San Diego, CA, USA) was used for statistical data analysis. Results were summarized as the mean ± SD values with standard deviations obtained from a minimum of three separate experimental runs. Differences between study groups were assessed using analysis of variance, which was supplemented by Dunnett’s post hoc test for multiple comparisons. Statistical significance was defined as *p* < 0.05.

### 2.5. Molecular Dynamics and Docking

#### 2.5.1. Structure Selection and Preparation

The structure of histone deacetylase 6 (HDAC6) was selected for in silico analysis. The structure was downloaded from the Protein Data Bank (PDB ID: 5EDU) [39]. Only the CD2 domain (residues Ser479–Arg835) containing a Zn^2+^ ion in the active site was selected for analysis. The inhibitor located in the active site was manually removed. A water molecule stabilizing the ion in the ligand-free structure was also added to the structure. The position of this water molecule was determined based on an analysis of the apo HDAC6 structure from Danio rerio (PDB ID: 5EEM). Hydrogen atoms were added to the structure at pH 7.4 using the H++ server [40]. His651 was protonated in the HIE tautomeric form (protonated at NE2 and lacking HD1 hydrogen) to allow coordination and stabilization of the zinc ion. The structure for molecular dynamics simulations was subsequently prepared using the AMBER MCPB.py [41] and LEaP [42] tools, which employed the TIP3P water model and the FF14SB force field. Geometry optimization was performed using Gaussian 09 (revision D.02) [43]. The residues His651, Asp649, and Asp742, as well as one water molecule, were designated as zinc-coordinating ligands. The ionic charge of the Zn atom was set to +2.

A short molecular dynamics simulation was performed using the AMBER 24 software package [44] to relax and equilibrate the protein structure prior to docking. The simulation was performed to generate a locally equilibrated starting conformation for subsequent in silico analyses. Energy minimization and equilibration were carried out following the protocol described by Daniel R. Roe and Bernard R. Brooks (“A protocol for preparing explicitly solvated systems for stable molecular dynamics simulations”) [45]. The tenth stage, corresponding to the production run, was extended from 1 ns to 10 ns. The system was heated gradually from 0 K to 300 K. Production molecular dynamics simulations were conducted with a 2 fs timestep using Langevin dynamics (collision frequency: 5 ps^−1^) at 300 K. Long-range electrostatics were calculated using the Particle Mesh Ewald (PME) method with a 9 Å cutoff. All bonds involving hydrogen atoms were constrained using the SHAKE algorithm. Pressure was maintained at 1 atm using the Monte Carlo barostat. Trajectory coordinates were recorded every 1 ps. The coordinates of the protein from the final frame were saved as a separate PDB file and used for subsequent analysis.

#### 2.5.2. Molecular Docking

Two tested compounds, compounds **5** and **8**, were docked to the last frame obtained in the molecular dynamics simulation. Docking was performed using AutoDock Vina 1.2.3 [46] together with AutoDockTools4 [47]. The centre and dimensions of the docking box were set in the vicinity of the active centre: size_x = 20 Å, size_y = 20 Å, size_z = 20 Å, centre_x = 42.57 Å, centre_y = 38.25 Å, and centre_z = 45.16 Å. The ligands were prepared using the mk_prepare_ligand.py script, while the receptor was prepared using mk_prepare_receptor.py from the Meeko/AutoDock Suite package. Docking was performed using the Vina scoring function, with the parameters set to exhaustiveness = 32 to thoroughly search the conformer space and nummodes = 10 to generate the ten most favourable positions for each ligand. All output files were saved in .pdbqt format, which is compatible with AutoDock Vina. Protein–ligand interactions were performed using the Protein-Ligand Interaction Profiler (PLIP) server [48].

## 3. Results and Discussion

### 3.1. Chemical Part

**Scheme 1:**
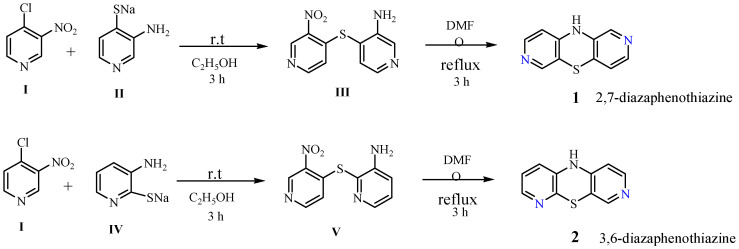
Synthetic pathways for the substrates necessary to obtain the final 10-heteroaryl dipyridothiazines [20,25].

In the first stage, the fundamental starting materials, 10H-2,7 and 3,6-diazaphenothioazines, were synthesized (compounds **1** and **2**). These compounds were obtained in a previously described multi-step synthesis using the corresponding derivatives of pyridine (**I**, **II**, and **IV**) and dipyridyl sulfides (**III** and **V**). These syntheses proceeded via the Smiles rearrangement and are shown in Scheme 1 [20,25].

**Scheme 2:**
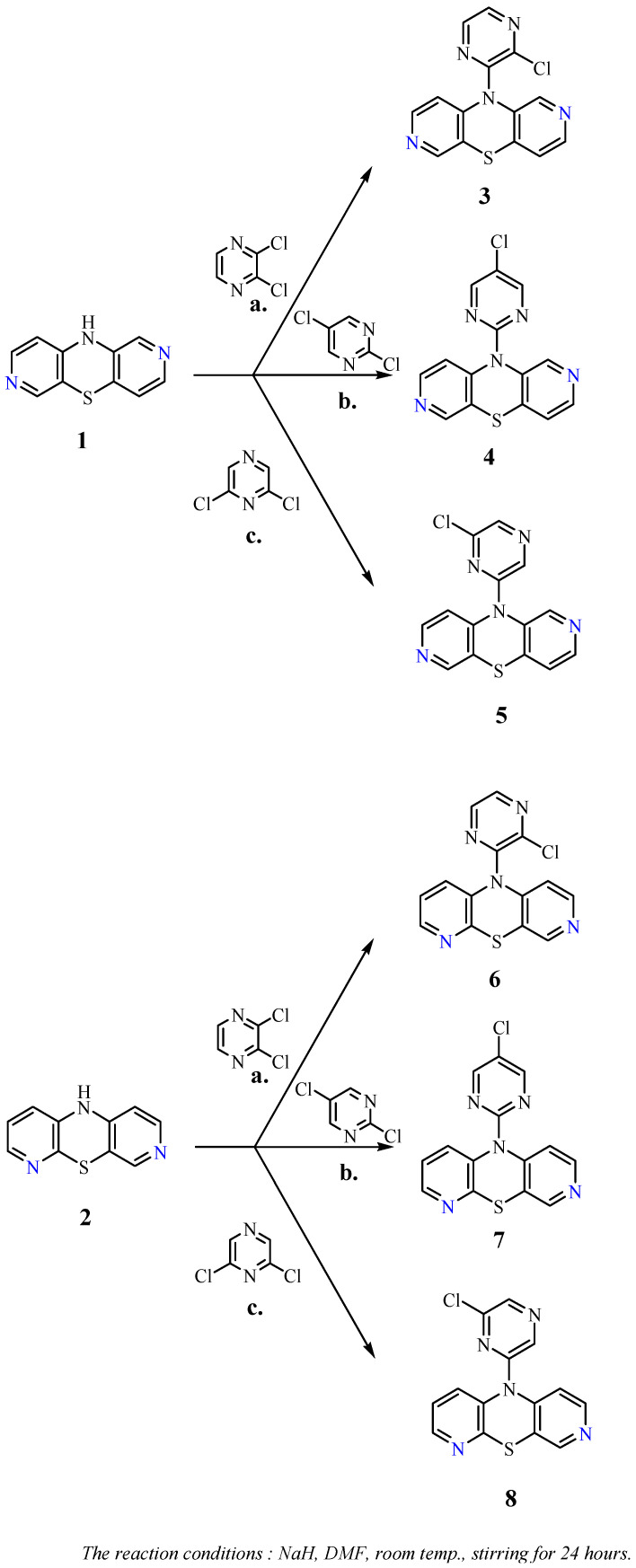
Synthesis of 10-heteroaryl dipyridothiazine derivatives **3**–**8**.

Next up, the dipyridothiazines were heteroarylated with selected dichloroazines: 2,3-dichloropyrazine (**a**), 2,5-dichloropyrimidine (**b**), and 2,6-dichloropyrazine (**c**). The reactions was performed in anhydrous DMF in the presence of the strong base NaH, leading to the formation of products **3**–**8** (Scheme 2). The reaction mixture was separated by column chromatography to obtain the final derivatives with good yields (80–91%). The obtained derivatives are isomeric compounds that differed not only in the location of nitrogen atoms in the dipyridothiazine core and in the heteroaryl substituent but also in the position of the chlorine atom in this substituent.

### 3.2. Structural Study

The structures of the new compounds were unambiguously confirmed by ^1^H and ^13^C NMR spectroscopy and high-resolution mass spectrometry (HRMS). ^1^H NMR spectra displayed the expected six aromatic protons of the dipyridothiazine core and two protons of the diazine substituent, while ^13^C NMR spectra corroborated the presence of all carbon atoms in the molecules. HRMS spectra consistently revealed isotopic peaks originating from the chlorine atoms in the molecule. HRMS analysis confirmed the correct molecular mass for all derivatives. All spectral analyses of new compounds are included in the Supplementary Materials.

### 3.3. Anticancer Activity Studies

In light of earlier promising results on the antiproliferative properties of dipyridothiazines [20,25,26,28], the cytotoxic potential of novel 10-heteroaryl derivatives was examined. The cytotoxic and antiproliferative properties of the novel derivatives were evaluated in vitro using the MTT assay on breast cancer (MDA-MB-231), lung cancer (A-549), and non-tumorigenic human keratinocyte (HaCaT) cell lines. The obtained results of the cytotoxicity analyses are included in Table 1.

Among these derivatives, compounds **3**–**5** exhibited moderate activity against breast cancer cells in the concentration range of IC_50_ = 28–87.7 μM but showed substantial cytotoxicity against normal human keratinocytes. The most active derivative in these studies was 2,7-diazaphenothiazine with a 6-chloropyrazyl substituent (compound **5**; IC_50_ = 28 μM). Compounds **3**–**5** also demonstrated cytotoxic activity against lung cancer cell lines, with activity in the IC_50_ = 9.6–67 μM range. As before, derivative **5** had the highest activity (IC_50_ = 9.6 μM), and it was additionally characterized by a significant selectivity index of SI = 3.45, which resulted from its low activity against normal cells. Analyzing the activity of chloroheteroaryl derivatives built on the 2,7-diazaphenothiazine core (derivatives **3**–**5**) indicates that the position of the chlorine atom in the azine substituent, as well as the location of the nitrogen atoms, had a significant impact on this activity. Derivatives with a pyrazine ring (derivatives **3** and **5**) were more active than those with a pyrimidine substituent (derivative **4**). Furthermore, analogous derivatives built on a 3,6-diazaphenothiazine core (derivatives **6**–**8**) showed no cytotoxic activity, which was unexpected compared to previous results [25]. A comparative evaluation of compounds **4** and **7**, which contain a 5-chloro-2-pyrimidyl substituent, with the previously described 10-(2′-pyrimidyl)-3,6-diazaphenothiazine [25] (Figure 1, derivative **B**), which was noted for its strong in vitro anticancer activity against MCF-7 breast cancer cells (IC_50_ = 0.73 μg/mL; 2.73 μM), suggests that chlorination of the pyrimidyl moiety has a detrimental impact on biological activity. The presence of the chlorine atom appears to significantly attenuate, or even abrogate, the antiproliferative potential of these derivatives, highlighting the sensitivity of this scaffold to subtle structural modifications. After comparing the obtained cytotoxicity results of the tested derivatives to the activity of the reference drugs (cisplatin and doxorubicin), it can be concluded that the group containing these derivatives showed rather weak activity, with the exception of compound **5**, but were relatively nontoxic to normal cell lines compared to the reference substances in the range of the tested concentrations.

### 3.4. Molecular Docking

To clarify the biological activity of compound **5** based on the 2,7-diazaphenothiazine scaffold and the inactivity of its isomer (compound **8**), which was built on 3,6-diazaphenothiazine, molecular docking was performed in relation to human histone deacetylase 6 (HDAC6) that was described as a molecular target [30,31,32]. Only the CD2 catalytic domain was selected for analysis, as it is the catalytically active domain responsible for inhibitor binding and is regarded as the primary target for small-molecule modulation of HDAC6 activity [34].

There is no ligand-free human HDAC6 structure in the PDB database. The selected structure (PDB ID: 5EDU) contains a bound trichostatin A inhibitor. For this reason, the conformation of the enzyme active site may differ from the apo form, making structural relaxation necessary prior to docking. To address this, a relaxation and equilibration step with short 10 ns production was performed in the first stage of the study to relax the system. RMSD and RMSF analyses (Supplementary Figure S1) were used to confirm the stability of the structure during MD simulations. RMSD values remained in the range of ~0.8–1.0 Å, indicating that the system quickly reached equilibrium and did not undergo significant conformational changes. Most amino acid residues showed low fluctuations (RMSF < 1 Å), with a few more flexible local fragments. The results confirm that the structure was effectively relaxed prior to the docking stage, ensuring a stable and representative initial conformation. Both compounds were docked in the ten most favourable positions. Despite the significant similarity in structure, many key differences for both ligands were observed during the docking analysis. Supplementary Table S1 summarizes the most important energy results.

**Figure 2:**
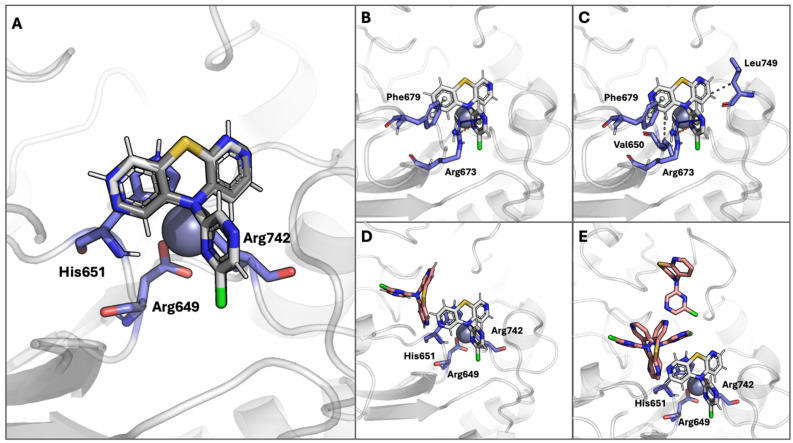
(**A**)—the lowest energy positions of compound **5** (white carbon atoms) and compound **8** (grey carbon atoms) in the binding pocket. (**B**,**C**)—interactions of compounds **5** and **8**, respectively, in the active site pocket. (**D**,**E**)—docking positions of poses marked as outliers for both compounds (red carbon atoms). Sticks with light blue carbon atoms indicate amino acids stabilizing the zinc ion. Structures were drawn with ChemDraw (Version 23.1.1.3). RevvitySignalsSoftware (2024), Waltham, MA, USA.

**Figure 3:**
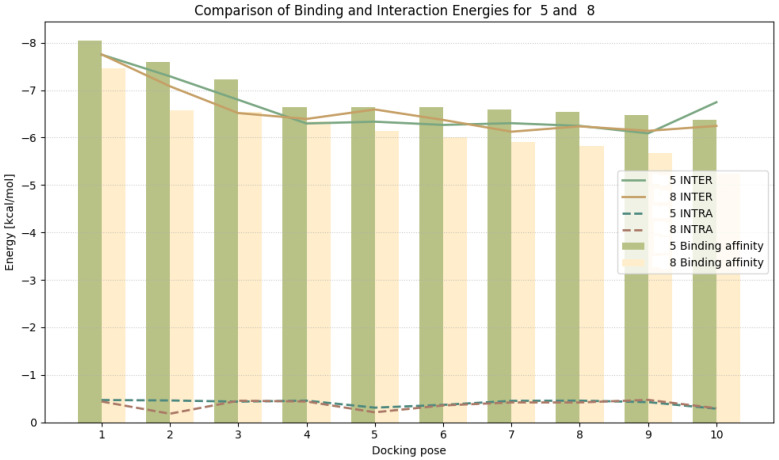
Energy results after docking for both compounds. The bars define binding affinity. Solid lines indicate ligand–protein interactions (INTER), and dashed lines describe ligand internal energy (INTRA).

Both ligands adopt the same binding position in the first pose, with an RMSD of 0.065 Å upon alignment (Figure 2A). This pose was identified as the most favourable for both ligands. Compound **5** exhibits a higher binding affinity (−8.038 kcal/mol) compared to compound **8** (−7.460 kcal/mol). Mean binding energies were −6.875 kcal/mol for compound **5** and −6.268 kcal/mol for compound **8**. Lower INTER energy value were also observed for compound **5**, suggesting stronger protein–ligand interactions. Ligand internal energy (INTRA) does not indicate unfavourable conformations, except for compound **5** at pose 10 (0.288 kcal/mol), which may suggest a slightly strained conformation. Overall, the energetic parameters indicate that compound **5** may have a higher affinity for HDAC6 than compound **8**, despite minimal structural differences (Figure 3).

In the subsequent steps, protein–ligand interactions were analyzed, focusing on the first docking poses, which had the lowest binding affinity values and were highly similar for both compounds. For both compounds **5** and **8**, three types of interactions with the same HDAC6 residues were detected: a hydrogen bond with Arg673, π-stacking with Phe679, and a π–cation interaction with Arg673 (Figure 2B). Additionally, compound **8** forms two additional hydrophobic contacts with Val650 and Leu749 (Figure 2C). Despite these additional interactions, compound **5** exhibits higher affinity for HDAC6, suggesting that the additional hydrophobic interactions in compound **8** are not energetically favourable and may destabilize the local protein structure to some extent. The analyses also assessed changes in ligand conformation across different poses by examining RMSD lower-bound (LB) and upper-bound (UB) values (Supplementary Table S2). These values reveal clear differences between the compounds. Compound **5** exhibits moderate conformational variability between poses, with an average RMSD LB of 3.362 Å and a single outlier at 7.137 Å (pose 5) (Figure 2D). In contrast, compound **8** shows substantially larger discrepancies in RMSD LB values, with an average of 4.120 Å and three pronounced outliers: 7.013 Å (pose 6), 11.086 Å (pose 7), and 6.131 Å (pose 9) (Figure 2E). A similar trend is observed in the RMSD UB analysis. Although RMSD UB values are generally higher, compound **5** demonstrates more consistent results (average: 5.347 Å; standard deviation: 2.694 Å) compared to compound **8** (average: 5.950 Å; standard deviation: 3.530 Å). Most docking poses, except for outliers, are located near the zinc ion. While both compounds **5** and **8** show some conformational variability across docking poses, compound **8** exhibits larger deviations and more pronounced outliers, suggesting that compound **5** adopts relatively more consistent binding poses in HDAC6.

To sum up the above analyses, it can be stated that both ligands, **5** and **8**, adopt virtually identical orientations in the HDAC6 pocket in the first pose, with an RMSD of 0.065 Å. Ligand **5** exhibits higher binding affinity than ligand **8**, as indicated by lower interaction energy values and more favourable complex energy. Conformational analysis further shows that compound **5** adopts more consistent and stable positions across different poses, whereas compound **8** displays greater variability and several pronounced RMSD deviations. Although compound **8** forms additional hydrophobic contacts, these do not compensate for the energetic advantage of compound **5**, which benefits from optimal key interactions and a better steric fit. Docking results reveal differences in the predicted binding modes of compounds **5** and **8**, although these findings alone cannot account for the marked contrast in their biological activity. The computational analysis therefore should be regarded as supportive rather than explanatory, offering structural context but not definitive structure–activity conclusions.

## 4. Conclusions

In conclusion, we have successfully synthesized a series of novel heteroaryl dipyridothiazines as structural isomers. The target compounds were efficiently obtained via heteroarylation of 2,7- and 3,6-diazaphenothiazines with selected dichloroazines, including 2,3-dichloropyrazine, 2,5-dichloropyrimidine, and 2,6-dichloropyrazine. These derivatives offer versatile scaffolds for further investigation of structure–activity relationships and hold promise for the development of biologically active agents. The chemical structures of the newly synthesized derivatives were unambiguously confirmed by NMR and HRMS spectroscopic analyses. The cytotoxic potential of the compounds was evaluated using the MTT assay against normal human keratinocytes (HaCaT) and two cancer cell lines—human breast adenocarcinoma (MDA-MB-231) and human lung carcinoma (A-549)—with cisplatin and doxorubicin as reference drugs. The activity of the tested derivatives depended on both the type of cancer cell line and the structure of the dipyridothiazine derivatives. Among the compounds tested, a promising derivative (derivative **5**) was identified, with an IC_50_ = 9.6 µM against lung cancer cells, which was characterized by a significant selectivity index. Notably, the conducted studies revealed that the isomeric derivatives built on the 3,6-diazaphenothiazine scaffold were biologically inactive. The consistent lack of activity observed for derivatives **6**–**8** prompted us to explore the underlying reasons for this behaviour, which was further investigated using molecular modelling complemented by molecular dynamics simulations. Molecular docking experiments performed for HDAC6 provided structural insights into the interaction patterns to derivative **5** and its isomer (compound **8**). Compound **5** exhibited more favourable predicted binding scores and a more coherent interaction network within the active site than compound **8**. Nevertheless, these computational results alone cannot account for the pronounced differences in their antiproliferative activity. The docking analysis therefore serves primarily as a complementary structural perspective, highlighting possible interaction preferences but not enabling firm conclusions regarding the underlying structure–activity relationships. Further studies will be required to clarify the molecular basis of the divergent biological profiles of these derivatives.

## Figures and Tables

**Table 1:** Cytotoxic activity (IC_50_, µM) of studied compounds estimated by the MTT assay ^a^.

Compounds	Cancer Cells				Normal Cells
	MDA-MB-231 *^d^*		A-549 *^e^*		HaCaT *^f^*
	IC_50_ *^b^*	SI *^c^*	IC_50_	SI	IC_50_
**3**	54.5 ± 2.14	0.34	31.7 ± 8.45	0.59	22.5 ± 7.46
**4**	87.7 ± 3.64	0.77	67.1 ± 16.69	1.01	58.7 ± 22.01
**5**	28.3 ± 8.68	1.17	9.6 ± 4.46	3.45	35.4 ± 5.23
**6**	99.3 ± 6.14	1.00	>100	1.00	>100
**7**	>100	1.00	>100	1.00	>100
**8**	>100	1.00	>100	1.00	>100
**Doxorubicin ^g^**	1.6 ± 0.23	0.19	0.2 ± 0.09	1.5	0.3 ± 0.1
**Cisplatin ^h^**	7.8 ± 0.98	0.81	3.2 ± 1.24	5.08	6.3 ± 0.7

^a^ Data are presented as the mean ± SD (n = 3). *^b^* IC_50_ (µM) represents the half-maximal inhibitory concentration determined after 72 h of cell exposure to the respective compound. ***^c^***: The selectivity index (SI) was calculated using the following formula: SI = IC_50_ for normal cells/IC_50_ for cancer cells. *^d^*: Human breast adenocarcinoma cell line (MDA-MB-231), *^e^*: human lung carcinoma cell line (A-549), and *^f^*: human immortalized keratinocyte cell line derived from adult skin (HaCaT). ^g,h^: The reference compounds. IC_50_ ranges from 0.1 to 10 (extremely strong), 11 to 20 (strong), 21 to 50 (moderate), and 51 to 100 (weak).

## Data Availability

The original contributions presented in this study are included in the article/Supplementary Material. Further inquiries can be directed to the corresponding author.

## Supplementary Materials

The following supporting information can be downloaded at https://www.mdpi.com/article/10.3390/cimb48020128/s1.
