# Supplementary material for: Design, Synthesis, Analysis, and Cytotoxicity of Novel Heteroaryl Derivatives of Dipyridothiazines
Source: Curr Issues Mol Biol. 2026 Jan 23;48(2):128. doi: 10.3390/cimb48020128 (PMC12940024; doi:10.3390/cimb48020128)
Supplement: Supplementary file 1 [file cimb-48-00128-s001.zip › cimb-4112883-supplementary.pdf]

Supplementary Material

Design, Synthesis, Analysis and Cytotoxicity of Novel Heteroaryl Derivatives of Dipyridothiazines

Emilia Martula[1,2], Paulina Strzyga-Łach[3], Marta Struga[3], Katarzyna Żurawska[4], Weronika Bagrowska[5], Anna Kasprzycka[6], Małgorzata Jeleń[2], Beata Morak-Młodawska[2]*

[1] Doctoral School of The Medical University of Silesia, Poland, d201074@365.sum.edu.pl

[2] Department of Organic Chemistry, Faculty of Pharmaceutical Sciences, The Medical University of Silesia, Jagiellońska 4, 41-200 Sosnowiec, Poland; bmlodawska@sum.edu.pl (B.M.M.); manowak@sum.edu.pl (M.J)

[3] Chair and Department of Biochemistry, Medical University of Warsaw, 02-097 Warsaw, Poland, paulina.strzyga-lach@wum.edu.pl (P.S.Ł), marta.struga@wum.edu.pl (M.S.),.

[4] Biotechnology Centre, The Silesian University of Technology, Krzywoustego Street 8, 44-100 Gliwice, Poland; katarzyna.hopko@polsl.pl (K.Ż)

[5] Tunneling Group, Biotechnology Centre, Silesian University of Technology, Krzywoustego 8, 44-100 Gliwice, Poland; weronika.bagrowska@polsl.pl (W.B)

[6] Department of Organic Chemistry, Bioorganic Chemistry and Biotechnology, Faculty of Chemistry, The Silesian University of Technology, Krzywoustego Street 4, 44-100 Gliwice, Poland, anna.kasprzycka@polsl.pl (A.K)

Content

1. ^{1}H NMR, ^{13}C NMR, HR MS of 10-(3-chloropyrazin-2-yl)-10H-dipyrido[3,4-b:3',4'-e][1,4]thiazine (**3**)
2. ^{1}H NMR, ^{13}C NMR, HR MS of 10-(2-chloropyrimidin-5-yl)-10H-dipyrido[3,4-b:3',4'-e][1,4]thiazine (**4**)
3. ^{1}H NMR, ^{13}C NMR, HR MS of 10-(6-chloropyrazin-2-yl)-10H-dipyrido[3,4-b:3',4'-e][1,4]thiazine (**5**)
4. ^{1}H NMR, ^{13}C NMR, HR MS of 10-(3-chloropyrazin-2-yl)-10H-dipyrido[2,3-b:4',3'-e][1,4]thiazine (**6**) 21
5. ^{1}H NMR, ^{13}C NMR, HR MS of 10-(2-chloropyrimidin-5-yl)-10H-dipyrido[2,3-b:4',3'-e][1,4]thiazine (**7**)
6. ^{1}H NMR, ^{13}C NMR, HR MS of 10-(6-chloropyrazin-2-yl)-10H-dipyrido[2,3-b:4',3'-e][1,4]thiazine (**8**)
7. **Figure S1.** RMSD and RMSF plots after 10 ns molecular dynamics simulation.
8. **Table S1** Summarises the most important energy results.
9. **Tabela S2**. RMSD lower bound (LB) and upper bound (UB) results for each docking position.

1. ^{1}H NMR, ^{13}C NMR, HR MS of 10-(3-chloropyrazin-2-yl)-10H-dipyrido[3,4-b:3',4'-e][1,4]thiazine (**3**)

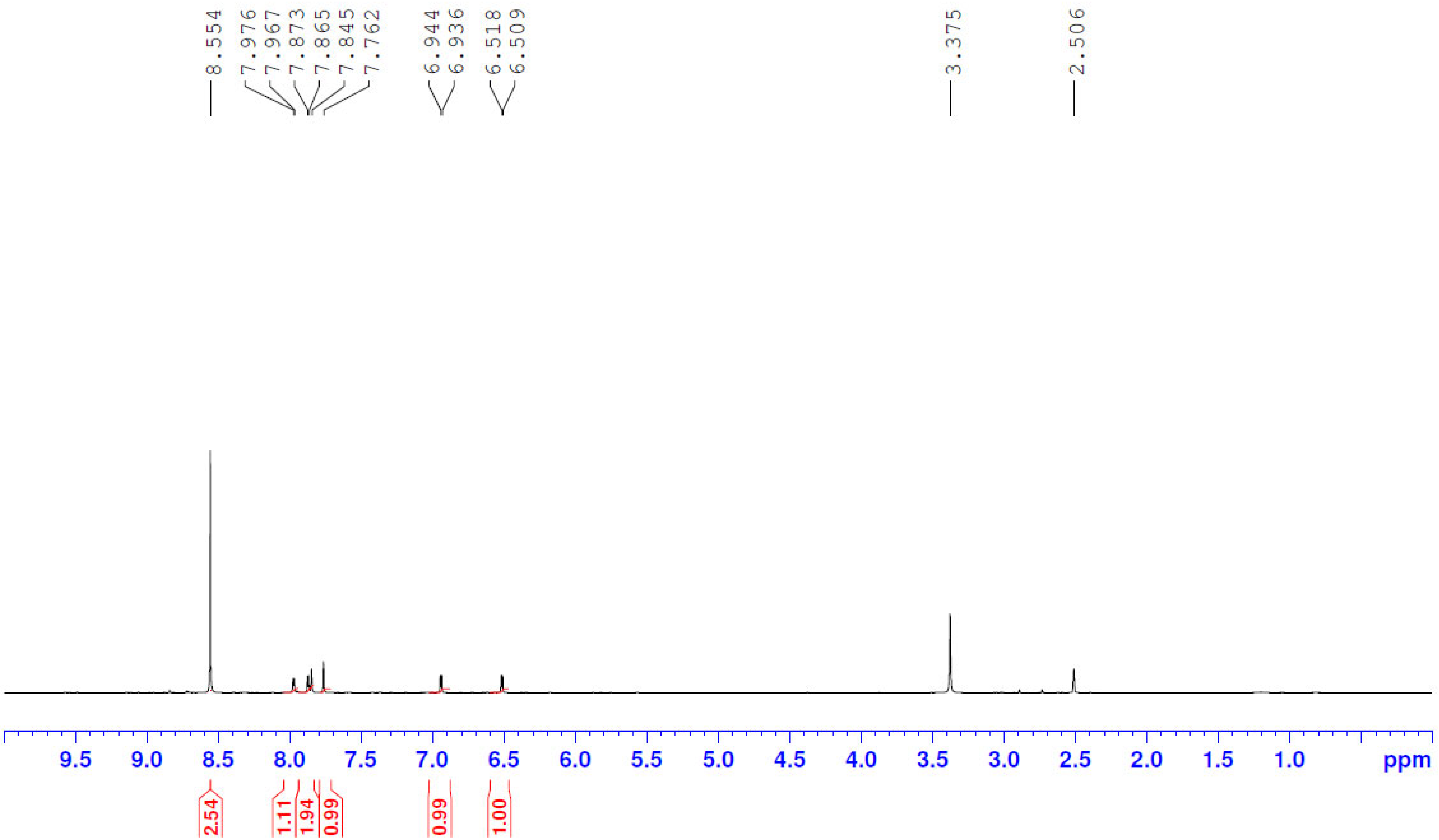

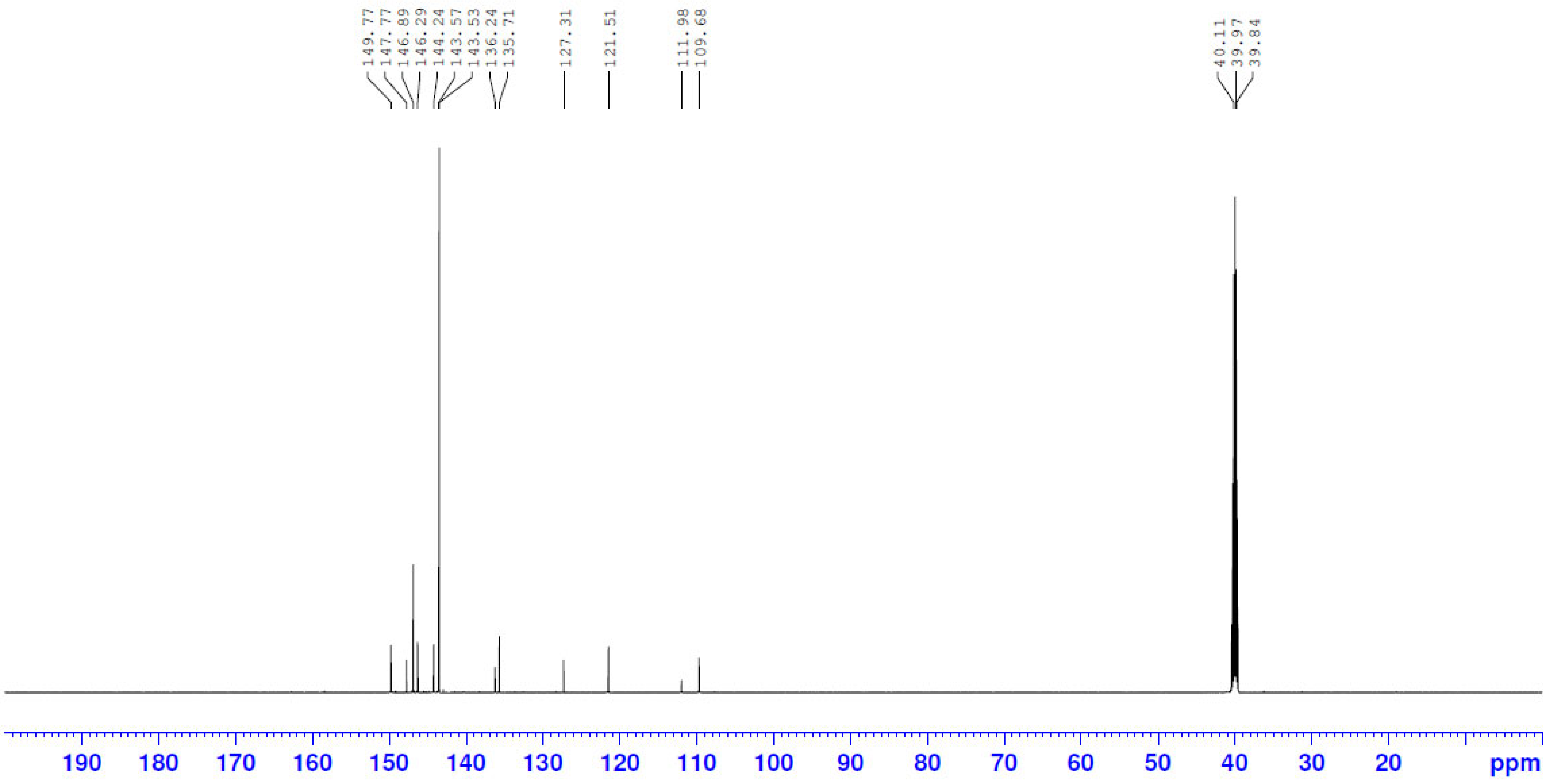
149.77
147.77
146.89
146.29
144.24
143.57
143.53
136.24
135.71
127.31
121.51
111.98
109.68
40.11
39.97
39.84
190
180
170
160
150
140
130
120
110
100
90
80
70
60
50
40
30
20
ppm

Acquisition Parameter

Source Type	ESI	Ion Polarity	Positive	Set Nebulizer	0.3 Bar
Focus	Active	Set Capillary	4000 V	Set Dry Heater	200 °C
Scan Begin	100 m/z	Set End Plate Offset	-500 V	Set Dry Gas	3.0 l/min
Scan End	1000 m/z	Set Charging Voltage	2000 V	Set Divert Valve	Source
		Set Corona	0 nA	Set APCI Heater	0 °C

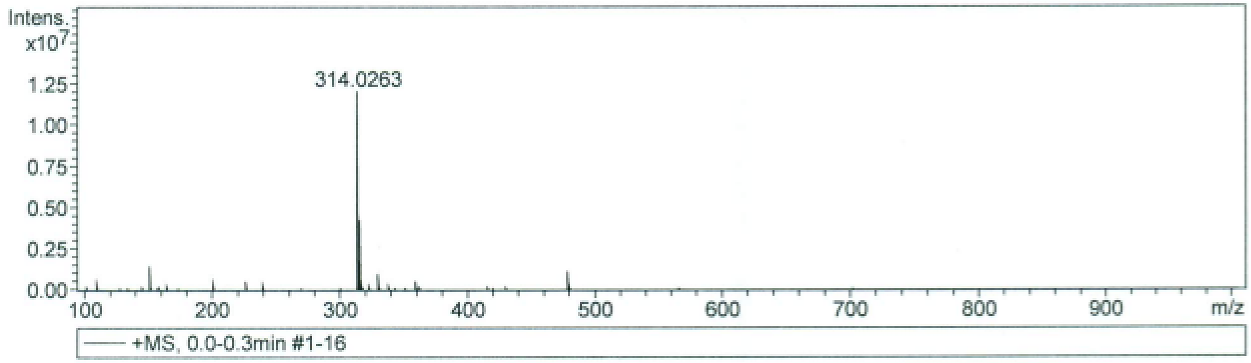

#	m/z	Res.	S/N	I	I %	FWHM
1	314.0263	39495	52482.0	12019570	100.0	0.0080
2	316.0235	37186	18412.8	4267461	35.5	0.0085

2. ^{1}H NMR, ^{13}C NMR, HR MS of 10-(2-chloropyrimidin-5-yl)-10H-dipyrido[3,4-b:3',4'-e][1,4]thiazine (**4**)

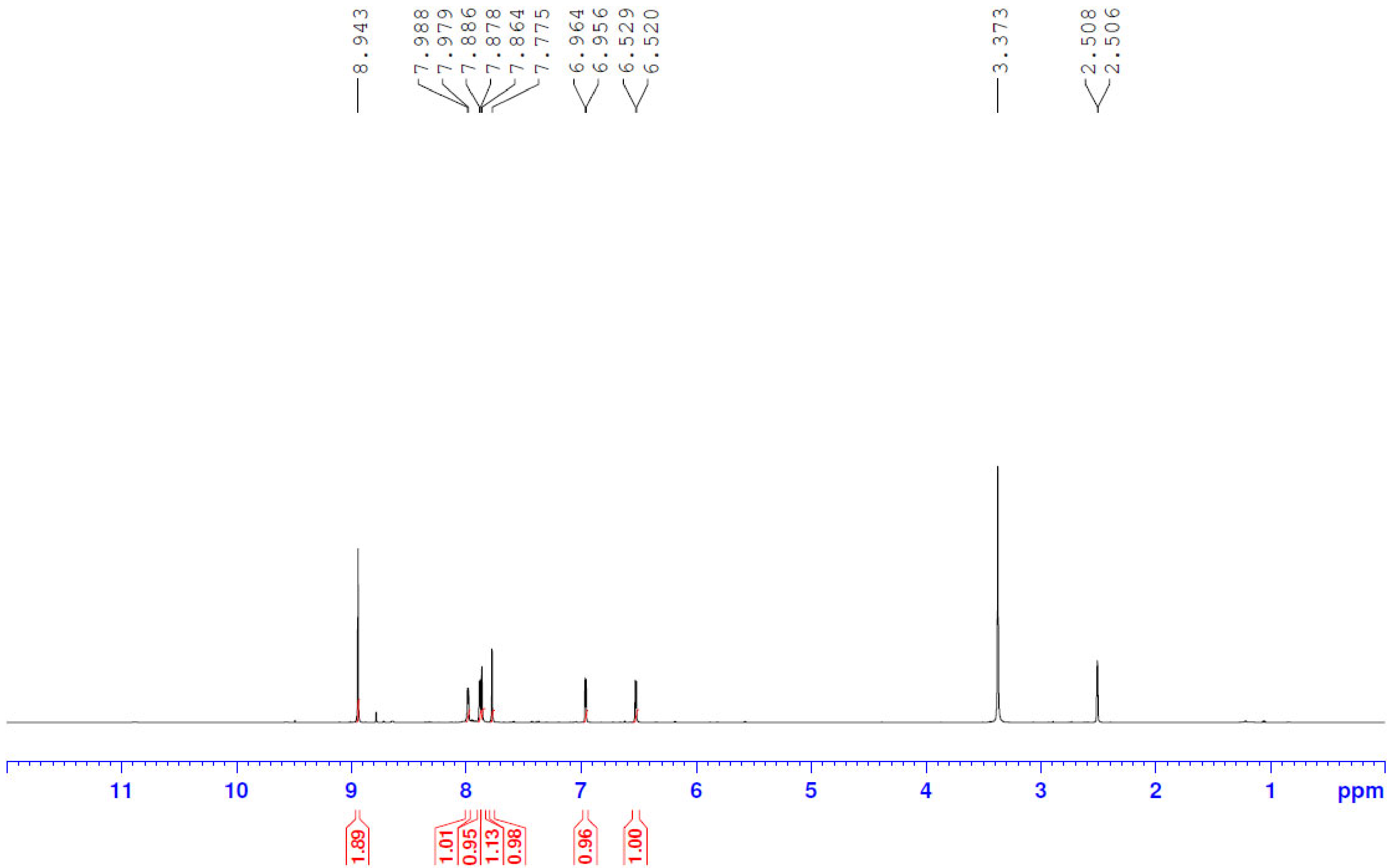

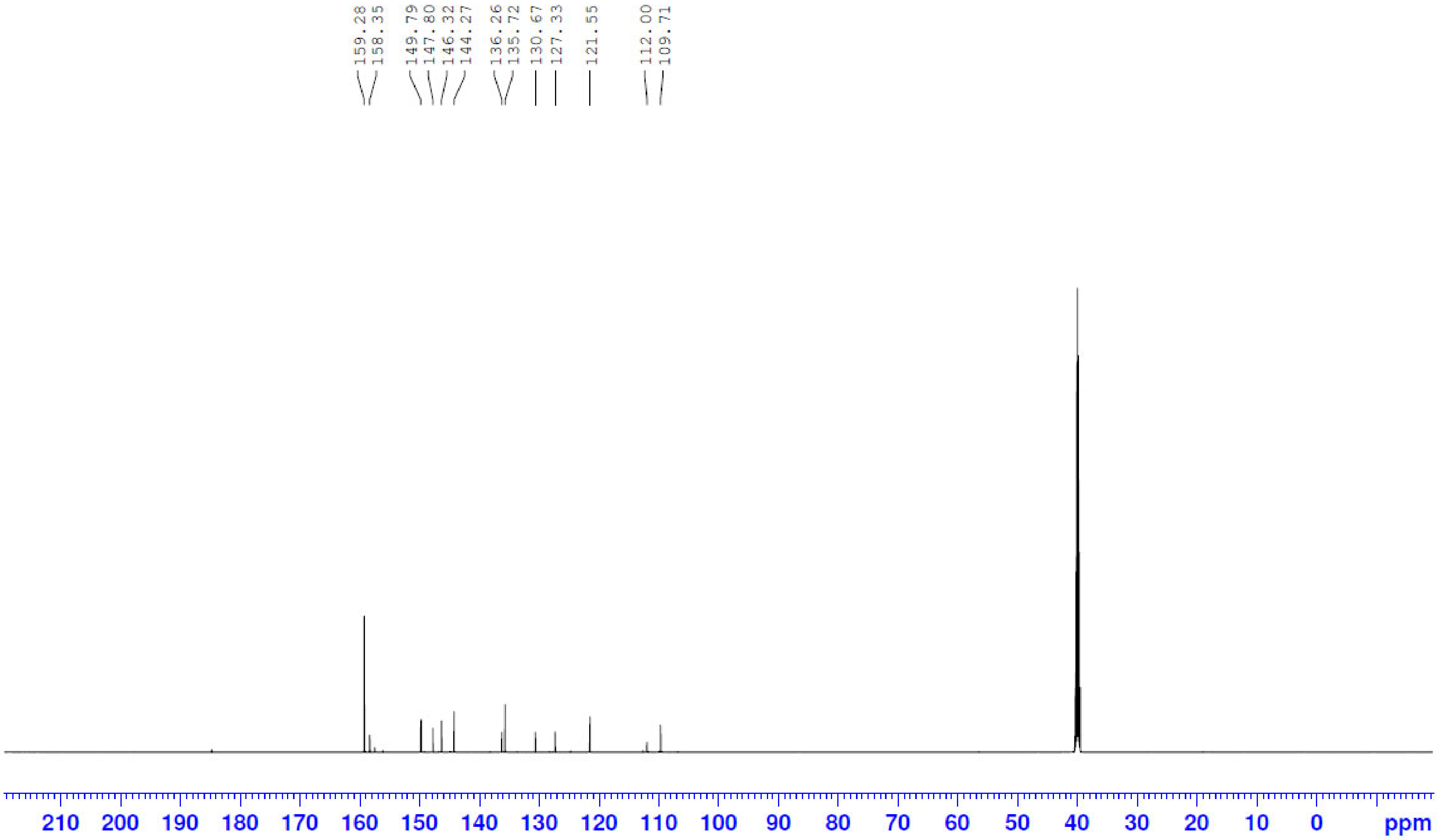

159.28
158.35
149.79
147.80
146.32
144.27
136.26
135.72
130.67
127.33
121.55
112.00
109.71
210 200 190 180 170 160 150 140 130 120 110 100 90 80 70 60 50 40 30 20 10 0 ppm

Acquisition Parameter

Source Type	ESI	Ion Polarity	Positive	Set Nebulizer	0.3 Bar
Focus	Active	Set Capillary	4000 V	Set Dry Heater	200 °C
Scan Begin	100 m/z	Set End Plate Offset	-500 V	Set Dry Gas	3.0 l/min
Scan End	1000 m/z	Set Charging Voltage	2000 V	Set Divert Valve	Source
		Set Corona	0 nA	Set APCI Heater	0 °C

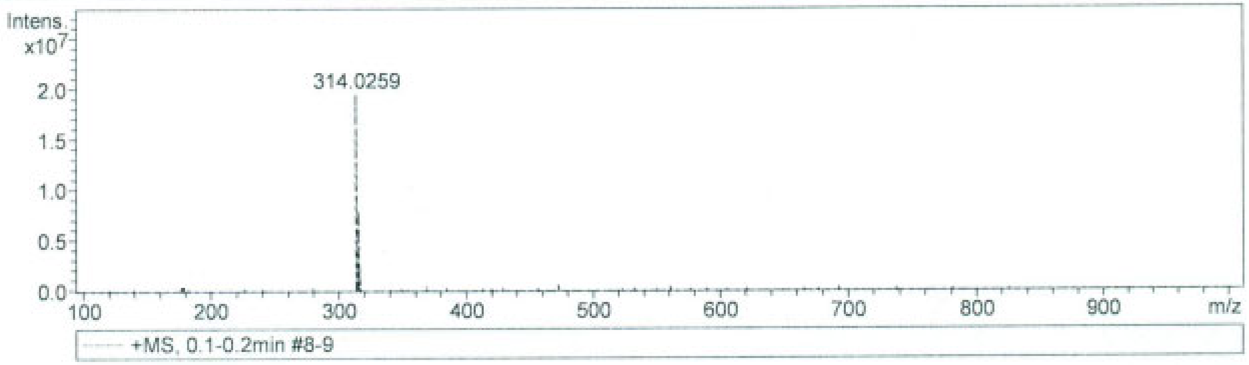

#	m/z	Res.	S/N	I	I %	FWHM
1	314.0259	27844	48993.9	19600658	100.0	0.0113
2	316.0228	38483	19841.5	8022451	40.9	0.0082

3. ^{1}H NMR, ^{13}C NMR, HR MS of 10-(6-chloropyrazin-2-yl)-10H-dipyrido[3,4-b:3',4'-e][1,4]thiazine (**5**)

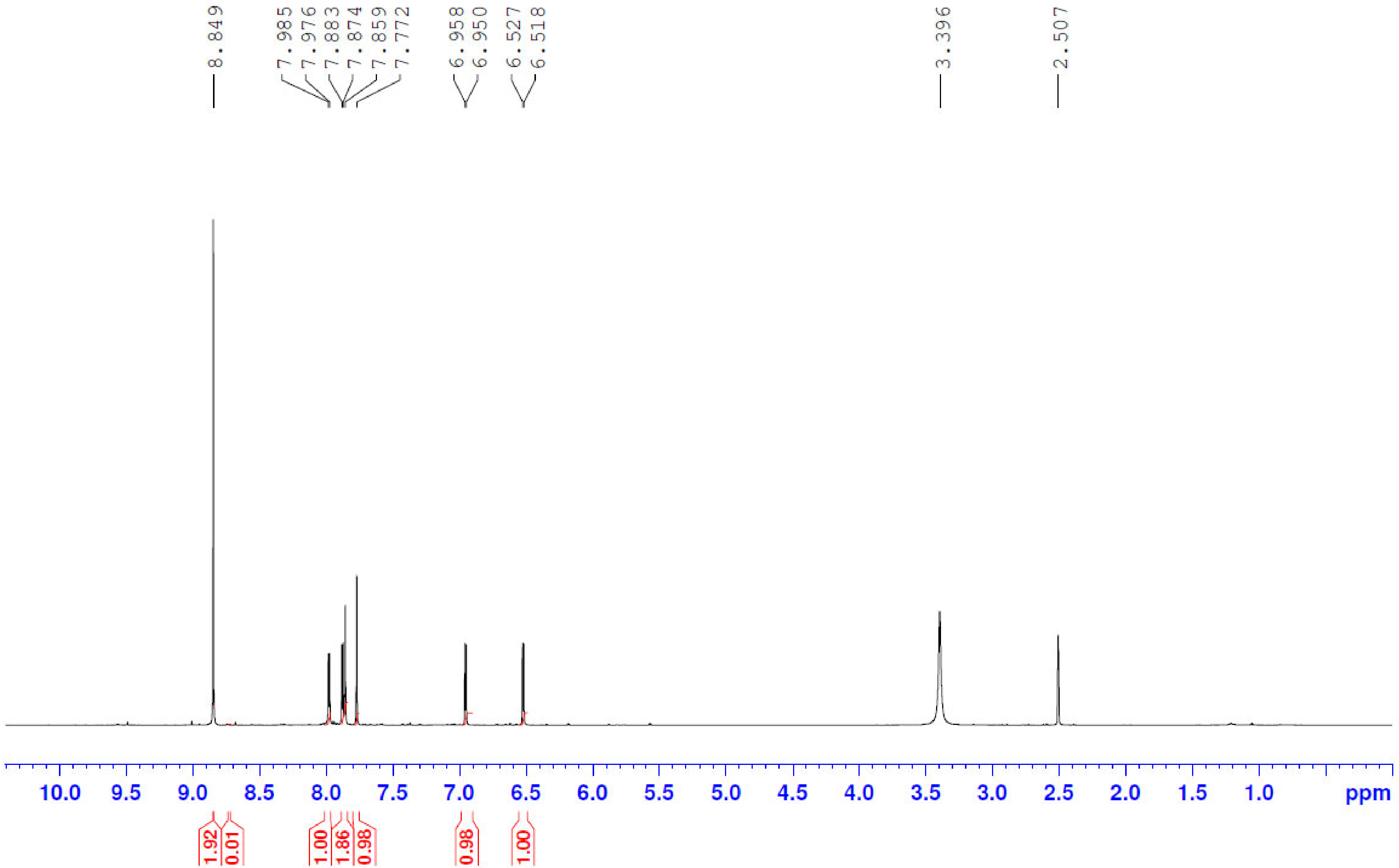

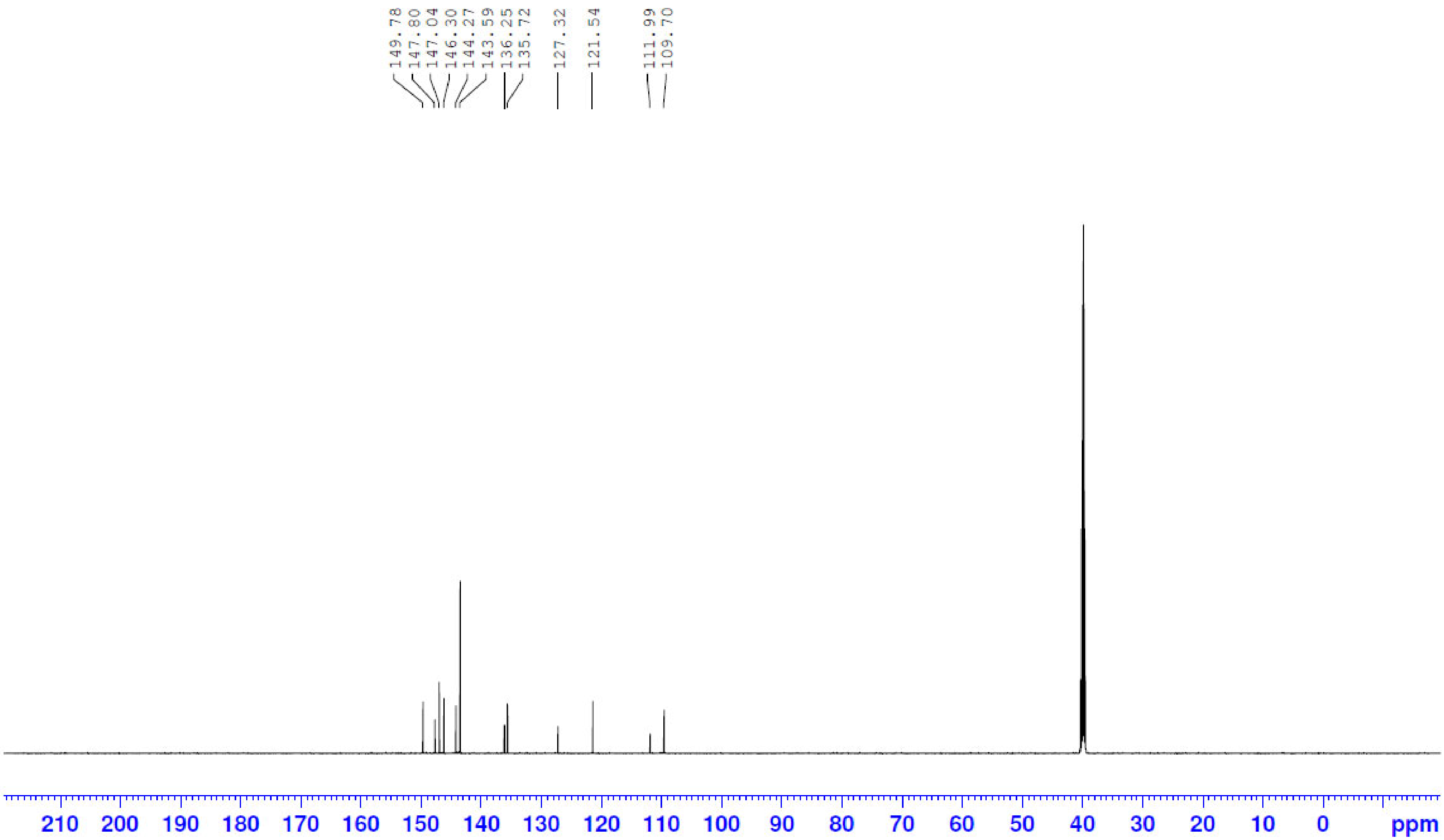
149.78
147.80
147.04
146.30
144.27
143.59
136.25
135.72
127.32
121.54
111.99
109.70
210 200 190 180 170 160 150 140 130 120 110 100 90 80 70 60 50 40 30 20 10 0 ppm

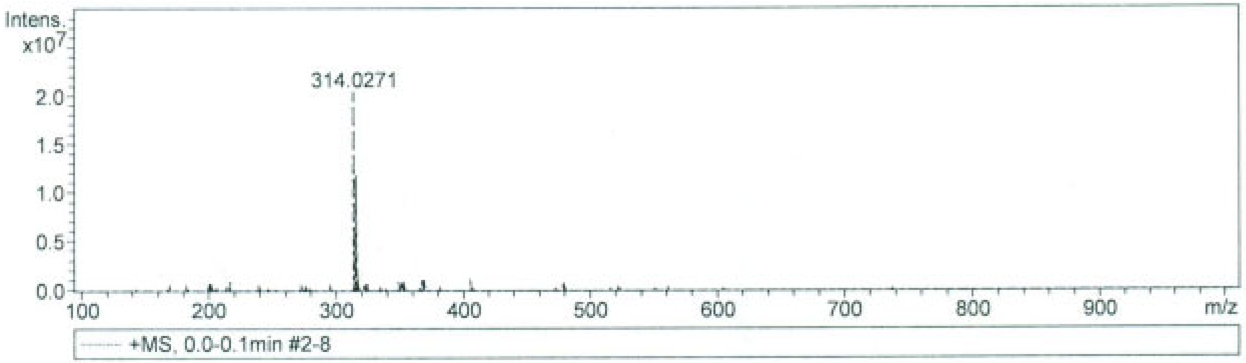

#	m/z	Res.	S/N	I	I %	FWHM
1	314.0271	17070	32338.3	20360036	100.0	0.0184
2	316.0232	39032	18580.6	11848672	58.2	0.0081

^{1}H NMR, ^{13}C NMR, HR MS of 10-(3-chloropyrazin-2-yl)-10H-dipyrido[2,3-b:4',3'-e][1,4]thiazine (**6**)

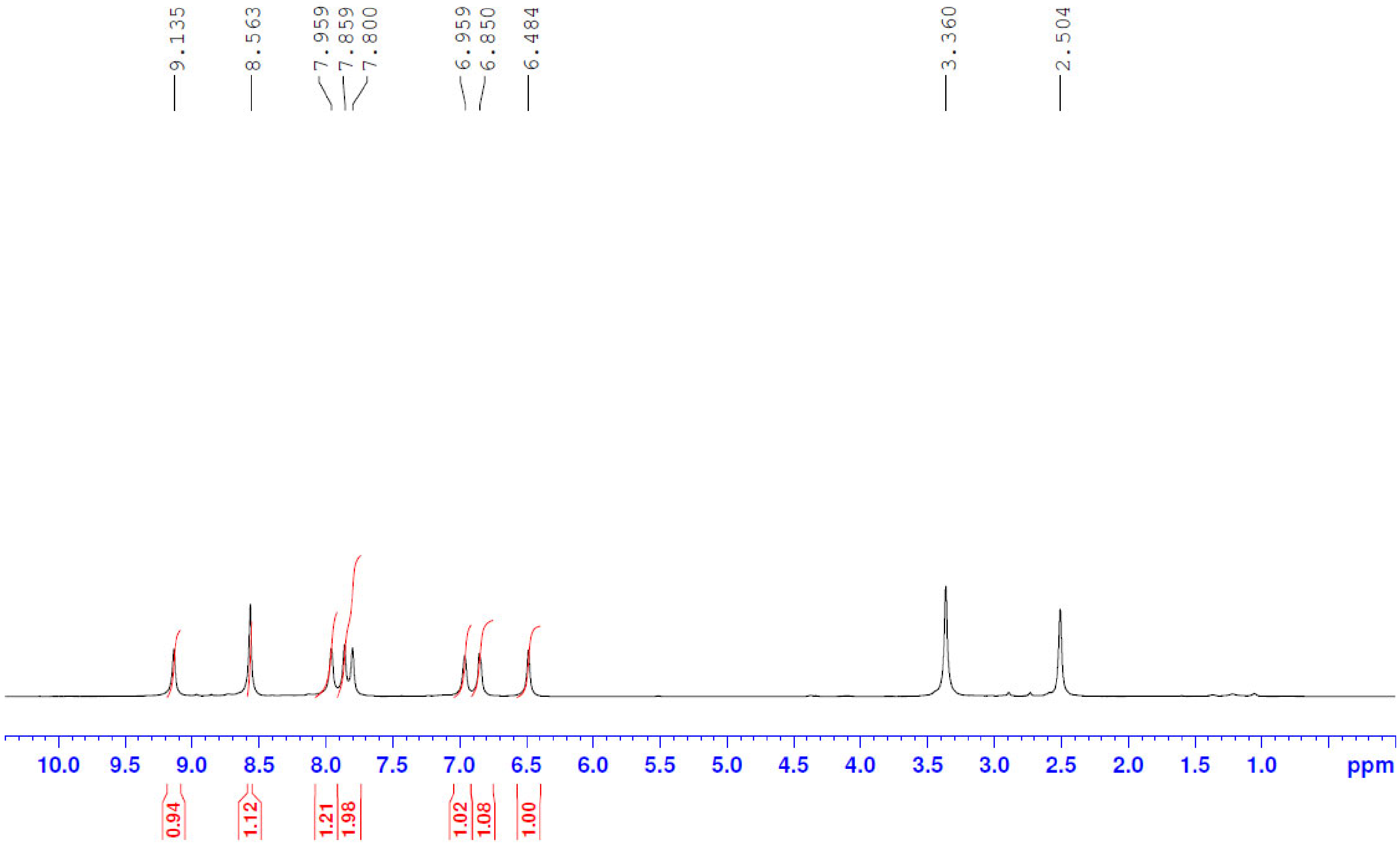

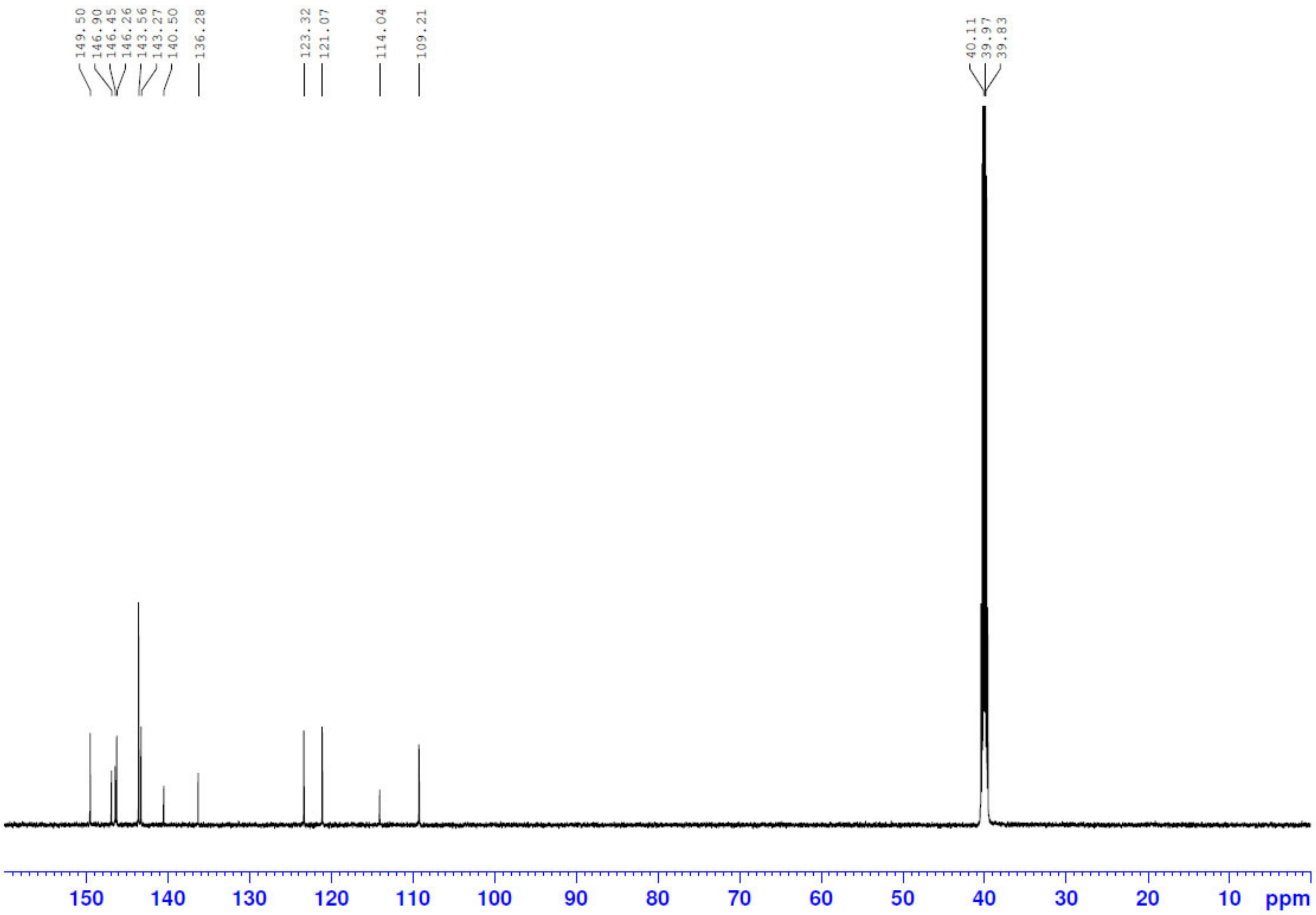
149.50
146.90
146.45
146.26
143.56
143.27
140.50
136.28
123.32
121.07
114.04
109.21
40.11
39.97
39.83
150
140
130
120
110
100
90
80
70
60
50
40
30
20
10
ppm

Acquisition Parameter

Source Type	ESI	Ion Polarity	Positive	Set Nebulizer	0.3 Bar
Focus	Active	Set Capillary	4000 V	Set Dry Heater	200 °C
Scan Begin	100 m/z	Set End Plate Offset	-500 V	Set Dry Gas	3.0 l/min
Scan End	1000 m/z	Set Charging Voltage	2000 V	Set Divert Valve	Source
		Set Corona	0 nA	Set APCI Heater	0 °C

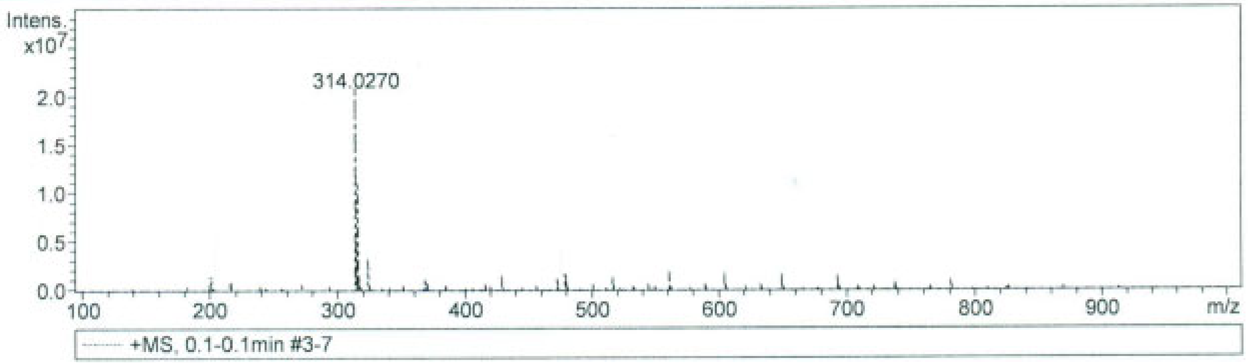

#	m/z	Res.	S/N	I	I %	FWHM
1	314.0270	17855	43538.3	20377592	100.0	0.0176
2	316.0231	39117	23569.2	11217862	55.0	0.0081

^{1}H NMR, ^{13}C NMR, HR MS of 10-(2-chloropyrimidin-5-yl)-10H-dipyrido[2,3-b:4',3'-e][1,4]thiazine (**7**)

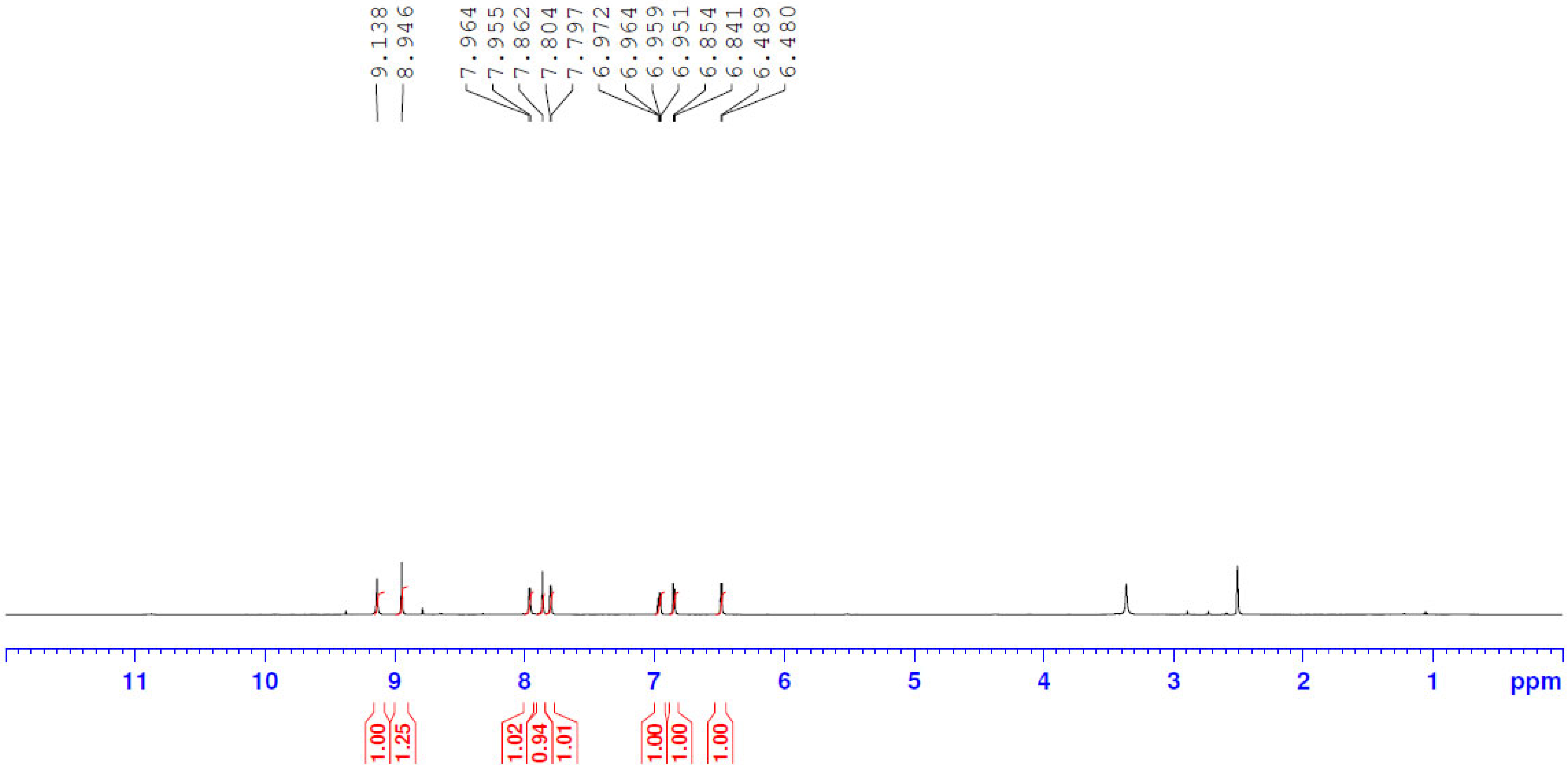

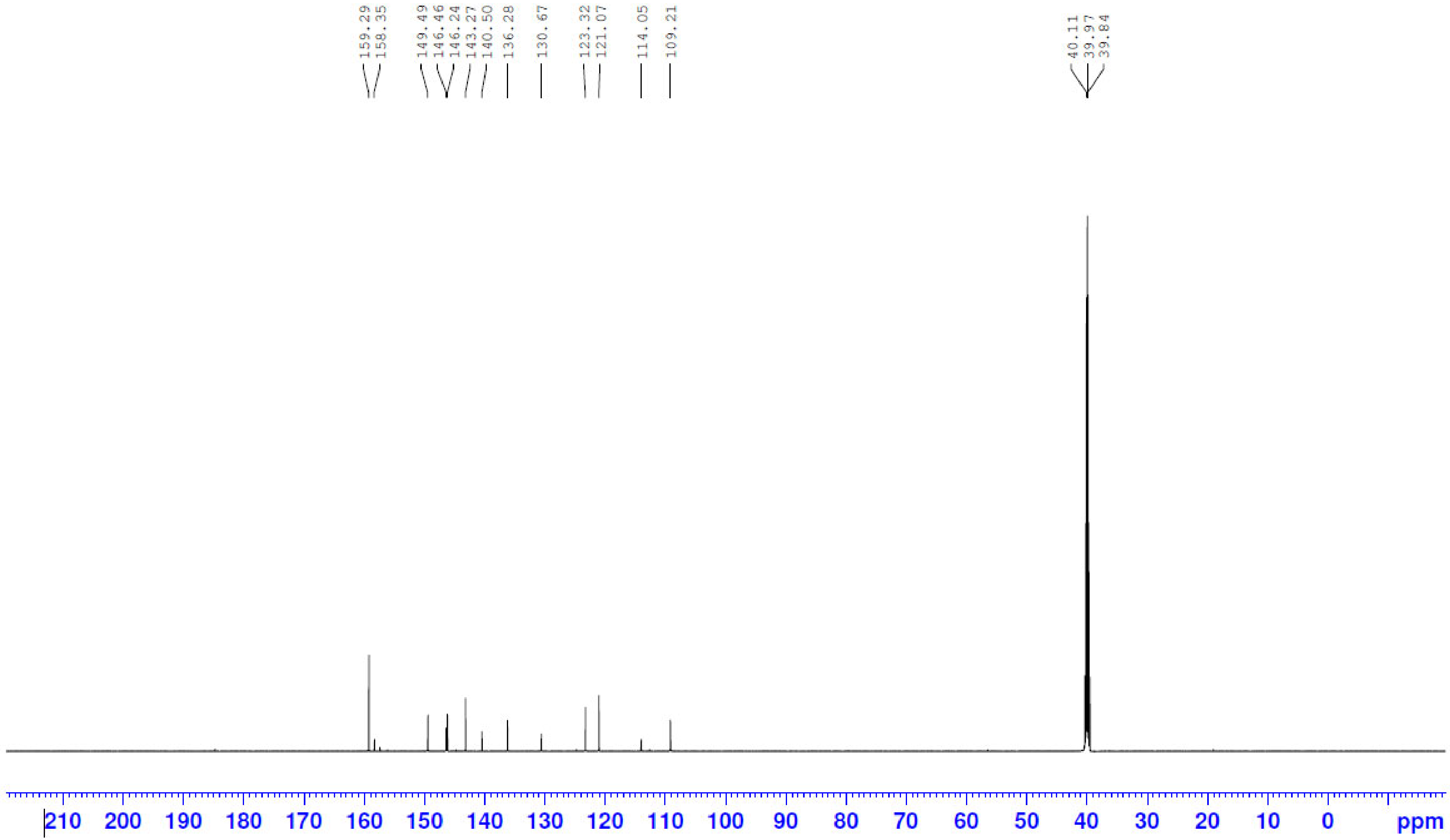
159.29
158.35
149.49
146.46
146.24
143.27
140.50
136.28
130.67
123.32
121.07
114.05
109.21
40.11
39.97
39.84
210 200 190 180 170 160 150 140 130 120 110 100 90 80 70 60 50 40 30 20 10 0 ppm

^{1}H NMR, ^{13}C NMR, HR MS of 10-(6-chloropyrazin-2-yl)-10H-dipyrido[2,3-b:4',3'-e][1,4]thiazine (**8**)

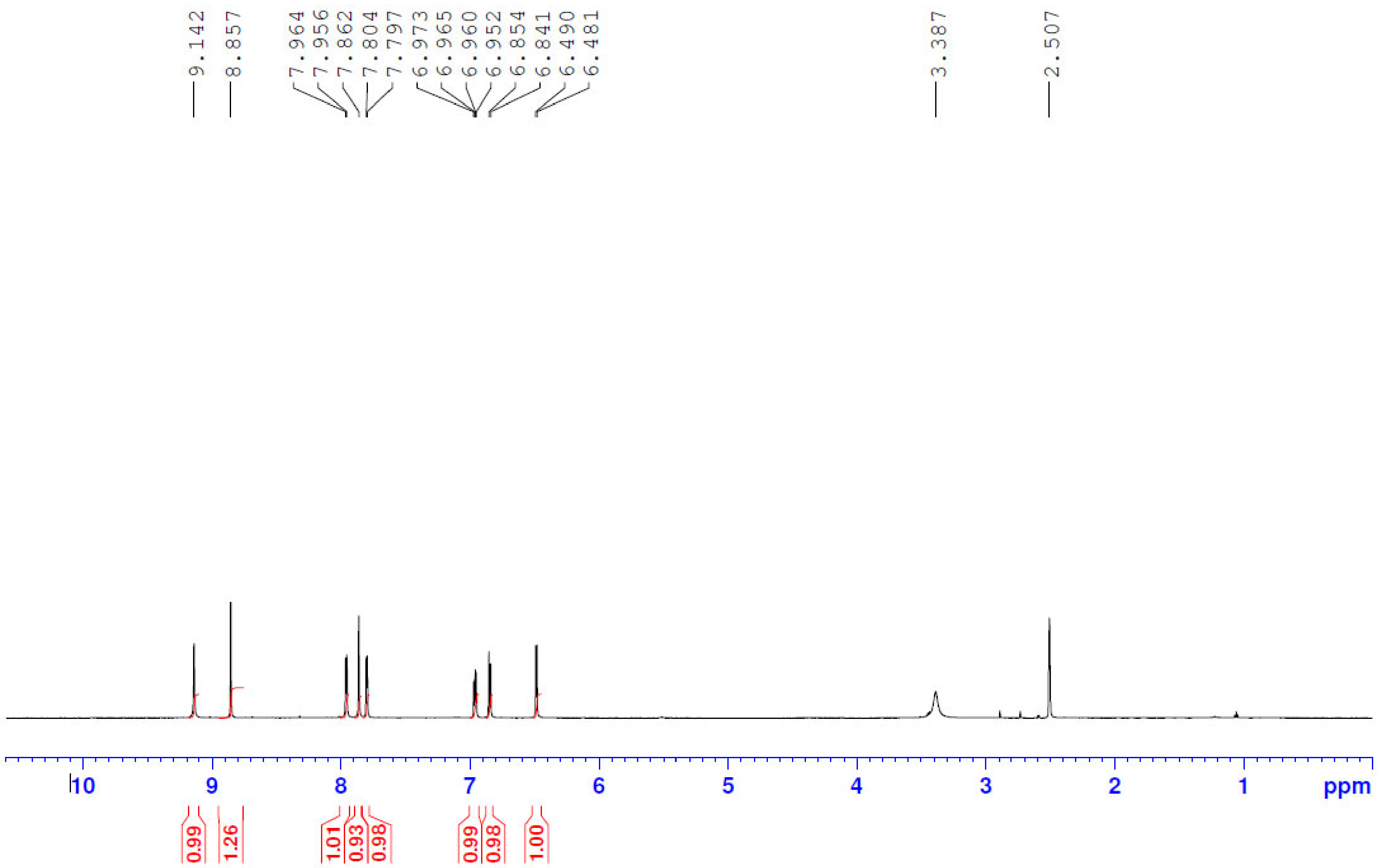

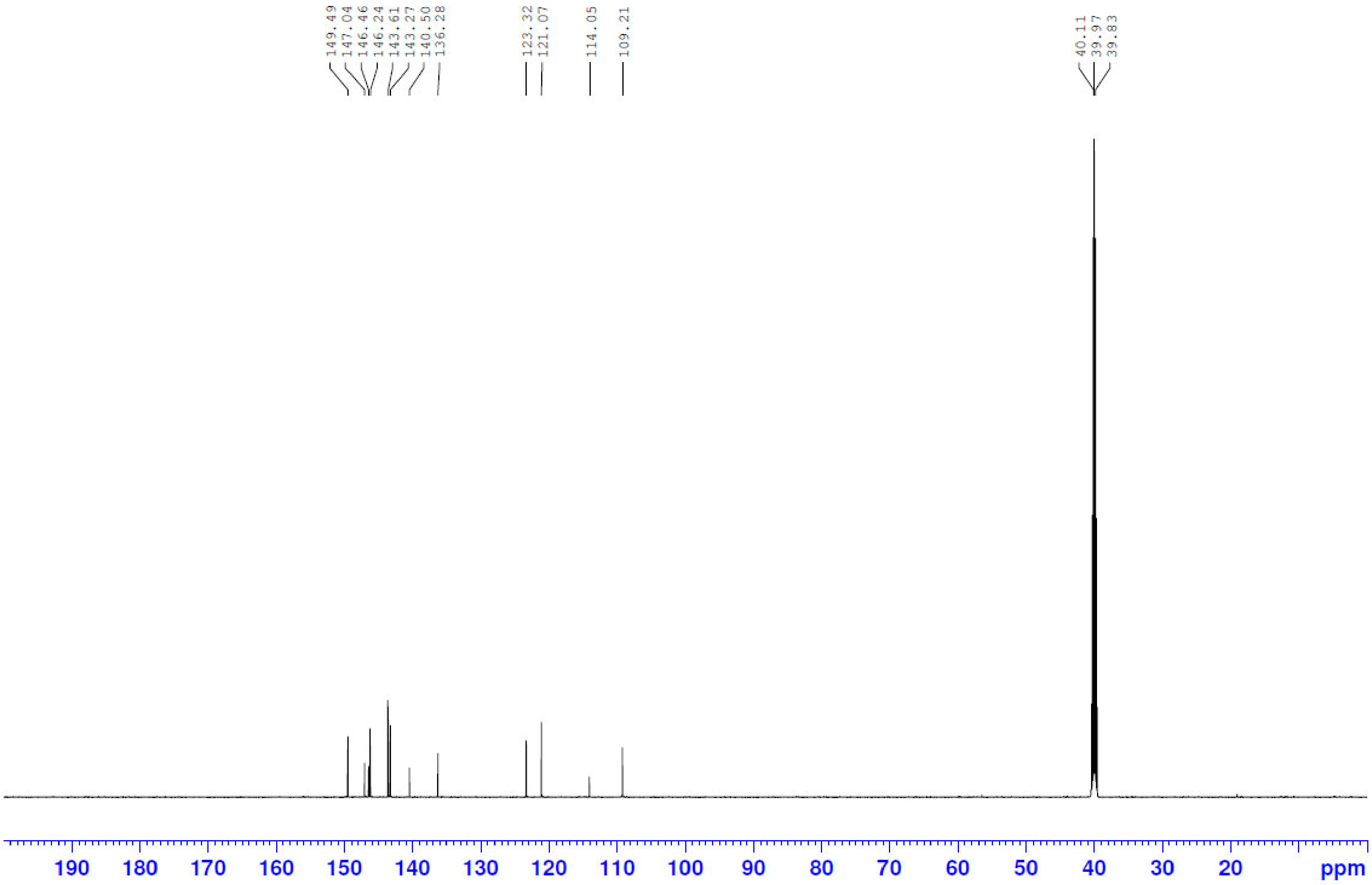
149.49
147.04
146.46
146.24
143.61
143.27
140.50
136.28
123.32
121.07
114.05
109.21
40.11
39.97
39.83
190
180
170
160
150
140
130
120
110
100
90
80
70
60
50
40
30
20
ppm

Acquisition Parameter

Source Type	ESI	Ion Polarity	Positive	Set Nebulizer	0.3 Bar
Focus	Active	Set Capillary	4000 V	Set Dry Heater	200 °C
Scan Begin	100 m/z	Set End Plate Offset	-500 V	Set Dry Gas	3.0 l/min
Scan End	1000 m/z	Set Charging Voltage	2000 V	Set Divert Valve	Source
		Set Corona	0 nA	Set APCI Heater	0 °C

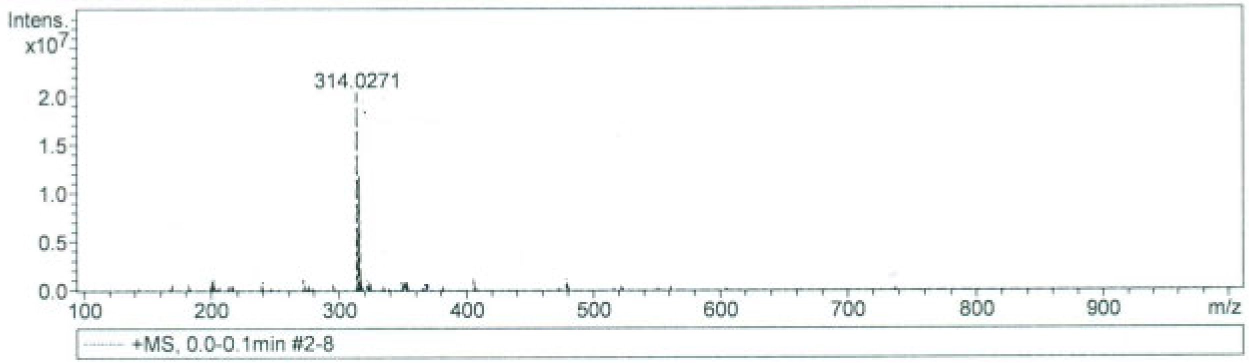

#	m/z	Res.	S/N	I	I %	FWHM
1	314.0271	17070	32338.3	20360036	100.0	0.0184
2	316.0232	39032	18580.6	11848672	58.2	0.0081

Figure S1. RMSD and RMSF plots after 10 ns molecular dynamics simulation.

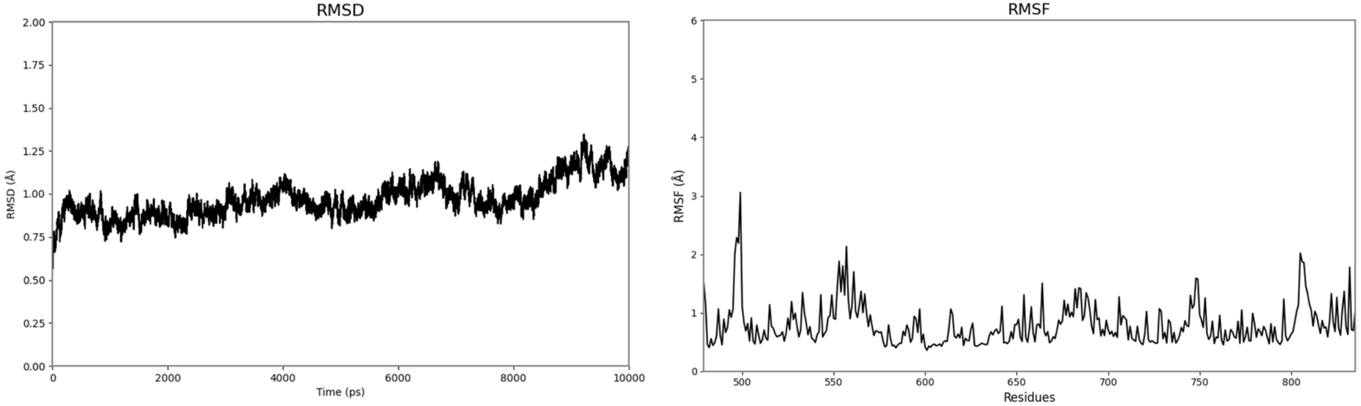

Table S1 Summarises the most important energy results.

The table contains the following energies: binding affinities, ligand-protein interactions (INTER), ligand internal (INTRA).

**5**				**8**			
**Docking pose**	**Binding affinity [kcal/mol]**	**INTER [kcal/mol]**	**INTRA [kcal/mol]**	**Docking pose**	**Binding affinity [kcal/mol]**	**INTER [kcal/mol]**	**INTRA [kcal/mol]**
**1**	-8.038	-7.749	-0.471	**1**	-7.460	-7.754	-0.440
**2**	-7.598	-7.292	-0.462	**2**	-6.580	-7.080	-0.182
**3**	-7.234	-7.109	-0.259	**3**	-6.353	-6.614	-0.408
**4**	-6.654	-6.297	-0.458	**4**	-6.155	-6.372	-0.440
**5**	-6.645	-6.335	-0.410	**5**	-6.148	-6.593	-0.212
**6**	-6.634	-6.269	-0.464	**6**	-6.106	-6.388	-0.372
**7**	-6.592	-6.231	-0.458	**7**	-6.057	-6.303	-0.406
**8**	-6.636	-6.367	-0.262	**8**	-6.018	-6.237	-0.430
**9**	-6.447	-6.089	-0.447	**9**	-5.902	-6.124	-0.420
**10**	-6.374	-6.746	0.288	**10**	-5.244	-6.244	-0.297
**MEAN**	**-6.875**	**-6.648**	**-0.340**	**MEAN**	**-6.268**	**-6.671**	**-0.361**
**SD**	**0.557**	**0.556**	**0.235**	**SD**	**0.466**	**0.497**	**0.096**

Tabela S2. RMSD lower bound (LB) and upper bound (UB) results for each docking position.

**5**			**8**		
**Docking pose**	**RMSD LB [Å]**	**RMSD UB [Å]**	**Docking pose**	**RMSD LB [Å]**	**RMSD UB [Å]**
**1**	0.000	0.000	**1**	0.000	0.000
**2**	1.237	1.522	**2**	2.798	5.102
**3**	2.926	5.112	**3**	4.055	6.211
**4**	2.889	5.031	**4**	1.479	1.736
**5**	7.137	9.766	**5**	3.073	5.367
**6**	4.226	7.446	**6**	7.013	9.661
**7**	4.313	6.246	**7**	11.086	13.083
**8**	3.088	5.439	**8**	2.648	4.984
**9**	4.356	7.307	**9**	6.131	7.979
**10**	3.441	5.347	**10**	3.878	5.339
**MEAN**	**3.362**	**5.347**	**MEAN**	**4.120**	**5.950**
**SD**	**1.823**	**2.694**	**SD**	**3.00**	**3.530**
